# Supplementary material for: Activity Regulation of a Glutamine Amidotransferase Bienzyme Complex by Substrate-Induced Subunit Interface Expansion
Source: ACS Catal. Author manuscript; Available in PMC 2025 May 13. (PMC7617670; doi:10.1021/acscatal.4c07438)
Supplement: Supplement [file EMS204876-supplement-Supplement.pdf]

Activity regulation of a glutamine amidotransferase bienzyme complex by substrate-induced subunit interface expansion

Franziska Jasmin Funke[1], Sandra Schlee[1], Isabel Bento[2], Gleb Bourenkov[2], Reinhard Sterner[1,*] and Matthias Wilmanns[2,3,*]

[1] Institute of Biophysics and Physical Biochemistry, Regensburg Center for Biochemistry, University of Regensburg, 93040 Regensburg, Germany

[2] European Molecular Biology Laboratory, Hamburg Unit, 22607 Hamburg, Germany

[3]University Hamburg Clinical Center Hamburg-Eppendorf, 20251 Hamburg, Germany

[$]Current address: European Molecular Biology Laboratory Grenoble, 38042 Grenoble Cedex 9, France

Corresponding authors:

Reinhard Sterner, Email: reinhard.sterner@ur.de

Matthias Wilmanns, Email: matthias.wilmanns@embl-hamburg.de

Supplement

Supplementary Table 1: X-ray crystallography data

**Protein**	**PabA/PabB**	**PabA/PabB**	**PabA/PabB**	**PabA/PabB**	**PabA**
**Chains**	A/D, B/C	A/D, B/C	A/D, B/C	A/D, B/C	A, B, C
**Added Ligands**		EDTA	Gln	Gln, Cho	
**Found Ligands**	Zn^{2+}, Trp	Trp	Gln-TE (A), Trp	Gln-TE (A), Cho, Trp	
**PDB entry**	8RP6	8RP2	8RP1	8RP0	8RP7
**Data collection**					
Space group	$P2_12_12_1$	$P2_12_12_1$	$P2_12_12_1$	$P2_12_12_1$	$P2_1$
Cell dimensions a, b, c (Å)	78.5, 108.3, 179.8	78.9 109.4 178.9	79.4, 109.9, 174.3	80.1, 109.9, 175.6	36.8, 115.6, 63.5
Resolution (Å)	2.45	1.98	1.86	1.64	2.82
R merge	0.147 (1.699)	0.103 (2.034)	0.099 (1.090)	0.120 (1.797)	0.313 (1.486)
$I/\sigma(I)$	11.9 (1.8)	16.7 (1.67)	16.7 (2.6)	11.2 (1.2)	6.8 (1.8)
CC 1/2	0.998 (0.806)	0.999 (0.693)	0.998 (0.853)	0.997 (0.677)	0.984 (0.513)
Completeness (%)	99.88 (99.79)	99.94 (99.95)	99.96 (99.98)	99.97 (99.99)	98.54 (98.31)
Redundancy	13.4 (13.5)	13.2 (13.6)	13.3 (13.5)	13.2 (13.3)	5.1 (5.1)
**Refinement**					
Resolution (Å)	46.39-2.45 (2.54-2.45)	44.9-1.98 (2.05-1.98)	46.88-1.86 (1.93-1.86)	46.6-1.64 (1.70-1.64)	42.39-2.82 (2.92-2.82)
No. reflections	57,801	108,389	128,391	189,813	12,411
R work / R free	0.204 / 0.254	0.200 / 0.239	0.167 / 0.201	0.169 / 0.199	0.211 / 0.268
**No. atoms**					
Protein chains	2 x PabA 2 x PabB	2 x PabA 2 x PabB	2 x PabA 2 x PabB	2 x PabA 2 x PabB	3 x PabA
Protein atoms	10166	10183	10194	10215	4423
Protein residues	1285	1285	1287	1286	567
Ligands	17	23	42	26	
Water	67	240	625	999	3
**B factors**					
Protein	63.2	50.9	36.7	35.8	56.1
Ligand	75.8	79.7	64.7	54.8	
Water	48.1	45.1	41.5	43.5	36.3
**R.m.s. deviations**					
Bond lengths (Å)	0.007	0.009	0.011	0.011	0.009
Bond angles (°)	1.50	1.54	1.56	1.67	1.41
**Ramachandran statistics (%)**					
Favored regions	96.5	96.9	97.4	97.3	92.7
Allowed regions	3.4	2.9	2.6	2.7	6.1
Outliers	0.2	0.2	0.0	0.0	1.3

The highest resolution shell values are in parenthesis.

Supplementary Table 2. Structural relations of representative ammonia-utilizing MST enzyme complexes

Enzyme	Organism	PDB entry	Rmsd [$Å^2$]	N_{res} [a]	N_{PabA} [b] [%]	$\Delta\varphi$ [c] [deg.]	Rmsd [$Å^2$]	N_{res} [a]	N_{PabB} [b] [%]	$\Delta\varphi$ [c] [deg.]
**ADCS**	*Escherichia coli*	8rp2 [d]	**PabA-based superposition**				**PabB-based superposition**			
	Streptomyces venezuelae	8hx6	1.18	170	91	19.6	1.46	405	89	19.6
**AS**	*Serratia marcescens*	1i7s	1.29	173	93	24.9	2.04	407	90	26.6
**AS**	*Salmonella typhimurium*	1i1q	1.17	175	94	24.9	1.99	403	89	27.9
**AS**	*Saccharolobus solfataricus*	1qdl	1.09	183	98	24.2	2.04	415	91	24.2
**ADICS**	*Burkholderia lata*	3r74	1.76	157	84	12.0	2.26	331	73	12

[a] Number of matching residues with the target sequence.

[b] Percentage of matching residues, using the PabA sequence (187 residues) or PabB sequence (455 residues) as reference.

[c] Change in orientation of PabB/synthase target principal axes resulting from a PabA-based superposition and PabA/synthase target principal axis resulting from a PabB-based superposition, calculated with PSICO (for further details see Materials and Methods, **Supplementary Figure 6**). Comparison of each value pair revealing very similar data demonstrates the robustness of the calculation of the principal domain/subunit axes by PSICO.

[d] ADCS coordinates of the glutaminase apo state (PDB ID: 8rp2) were used. All target structures were determined in the absence of glutaminase substrate, inhibitors or reaction ligands. The analysis demonstrates for representative structures of ammonia-utilizing MST enzyme complexes that despite close structural relationships at the level of separate glutaminase and synthase subunits/domains there is a substantial degree of variation in the orientation between the respective glutaminase and synthase domains/subunits, making an overall superposition of complete enzyme complexes unreliable or even impossible. Conformational changes at different catalytic states, as

performed elsewhere in this contribution for different ADCS complexes, were not considered in this comparative analysis of members of the MST family.

Supplementary Table 3: Gln-TE mediated PabA/PabB interface expansion and ADCS complex rearrangement

PDB entry	Gln-TE [a]	Cho/ Mg^{2+} [a]	Zn^{2+}, [a]	$\Delta\varphi$ [°] [c]	ASA [$Å^2$] [d]	N [d]	ΔG_{calc} [kcal/Mol][d]	$K_{D(calc)}$ [μM] [e]	$K_{D(calc, apo)}$ / $K_{D(calc, ligand)}$
8RP6			+		904	10	-7.7	2.2	
8RP2 (EDTA)					965	14	-8.1	1.1	
8RP1 (Gln [b])	+			23.4	1312	28	-10.6	0.017	129
8RP0 (Gln, Cho [b])	+	+		23.2	1324	27	-10.3	0.033	66

[a] Ligand observed in the electron density of the crystal structure

[b] Ligand added during crystallization

[c] Change in orientation of PabB principal axis calculated with PSICO, resulting from PabA-based superposition and using ADCS coordinates with no reaction ligands added (8RP6) as reference (**Supplementary Figure 6**). Since the PabA/PabB arrangement in 8RP6 and 8RP2 (ADCS coordinates with EDTA added) is identical, the $\Delta\varphi$ angles are unchanged when 8RP2 is used as the reference.

[d] ASA, accessible surface area; N, number of specific interactions between PabA and PabB subunits (hydrogen bonds, salt bridges), ΔG_{calc}, solvation energy gain, as defined in PDBePISA, version 1.48.

[e] $K_{D(calc)}$ values were calculated by applying $\Delta G_{calc} = RT * \ln K_{D(calc)}$

Supplementary Table 4: Effects of PabA/PabB active site ligands on ADCS steady-state glutaminase activity [a]

0.2 mM Cho	5 mM Mg^{2+}	0.2 mM Zn^{2+}	1 mM EDTA	K_M^{Gln} [mM]	k_{cat} [s^{-1}]	k_{cat}/K_M^{Gln} [$mM^{-1}s^{-1}$]
–	–	–	–	0.09 ± 0.005	0.20 ± 0.003	2.35 ± 0.17
–	+	–	–	0.09 ± 0.01	0.22 ± 0.007	2.41 ± 0.47
–	–	–	+	0.08 ± 0.002	0.19 ± 0.001	2.42 ± 0.08
–	+	–	+	0.09 ± 0.005	0.23 ± 0.003	2.58 ± 0.17
+	–	–	–	0.07 ± 0.005	0.14 ± 0.003	1.98 ± 0.18
+	+	–	–	0.34 ± 0.03	0.37 ± 0.010	1.09 ± 0.13
+	+	+	–	0.31 ± 0.008	0.42 ± 0.003	1.33 ± 0.04
+	–	–	+	0.08 ± 0.002	0.15 ± 0.001	1.91 ± 0.07
+	+	–	+	0.27 ± 0.010	0.31 ± 0.003	1.13 ± 0.05
**+**	+	+	+	0.31 ± 0.0n8	0.38 ± 0.006	1.24 ± 0.09

To investigate the role of zinc ion binding in ADCS catalysis we additionally purified the ADCS complex under conditions in which 0.2 mM $ZnCl_2$ was added to the expression medium to ensure an excess of zinc in the purified protein, since the addition of Zn^{2+} to the purified ADCS complex led to its precipitation. To measure ADCS glutaminase activity of these samples in the absence of Zn^{2+}, samples were dialyzed against a buffer containing EDTA. We also measured ADCS glutaminase activity in the presence and absence of synthase substrate Cho and Mg^{2+}**(Supplementary Figure 3a, Table 1)**. To this end, we could confirm previously established moderate changes on ADCS glutaminase activity by the presence of both Cho and Mg^{2+}, leading to a three to fourfold increase in K_M^{Gln} and an approximately twofold increase in k_{cat} of the ADCS glutaminase turnover [1]. These effects together resulted in a slight decrease in the ADCS glutaminase catalytic efficiency as measured by k_{cat}/K_M^{Gln}. In contrast, we found no significant change in ADCS glutaminase activity, caused by the either enforced presence of Zn^{2+} through its addition to the expression medium or its removal by EDTA treatment on ADCS glutaminase activity. These results demonstrate that, despite the close proximity of the ADCS Zn^{2+} binding site to the glutaminase active site, the presence of Zn^{2+} does not play a role in ADCS glutaminase catalysis.

Supplementary Table 5. PabA residues contributing to PabA/PabB interface, Zn^{2+} and Gln-TE binding

Residue	MC/SC [a]	IA [b]	Gln-TE [c]	Zn^{2+}, [c]	PabB, generic PabA/PabB interface	PabB, extended PabA/PabB interface
aS10	SC	HB				bK372
aT12	MC	HB			(bN312)	bN312
aW13	MC	HB			bN312	bN312
aN14	SC	HB			bD308	bD308
aQ17	SC	HB			bG315<br>bA318<br>(bG321)	bG315<br>bA318<br>bG321
aY18	SC	HB				bR311
aR30*	SC	SB				bE119
aG52	MC	HB	X			bY210
aC54*	MC	HB	X			bG207
aC79	SC	CB	X			
	SC			X		
aL80	MC	HB	X			
aQ83	SC	HB	X			
aK103	SC	SB			bD430	bD430
aY127*	SC	SB			 bD308	bL304<br>bD308
aH128*	SC			X		
aS129	SC	HB				bL204
	MC	HB	X			
aL130	MC	HB	X			
aH168	SC			X		
aE170	MC	HB			bR311	bR311
aS171*	SC	HB			bD308	bD308
aI172	MC	HB			bD308	bD308
Gln-TE	SC	HB				
	MC	HB				bG207<br>bY210

Asterisks: residues, which have been mutated for biophysical and biochemical characterization.

Parentheses: Interactions not detected in all relevant structures.

[a] MC, main chain; SC, side chain

[b] IA, interaction; HB, hydrogen bond; SB, salt bridge. The interactions have been determined as defined in PDBePISA, version 1.48.

[c] A distance cutoff of 3.5 Å was applied.

Supplementary Table 6: Glutaminase activity regulation in MST GATs

	ADCS	AS	ADICS
Glutaminase/Synthase interface expansion	x	x	x
Glutaminase/Synthase rearrangement	x		
Glutaminase active site Zn^{2+} binding	x		x
Synthase Trp binding site	x	x	
Trp feedback inhibition		x	

Supplementary Table 7. PabB residues contributing to Cho, Mg^{2+} and Trp binding

Residue	MC/SC [a]	IA [b]	Cho [c]	Mg^{2+} [c, d]	Trp [c]
bH35	MC	HB			x
bS36	SC	HB			x
bY43	MC	HB			x
bR45	MC	HB			x
bF46	MC	HB			x
bP240	MC	HB			x
bS242	MC	HB			x
bG275	MC	HB	x		
bT276	SC	HB	x		
	MC	HB		x	
bK298	SC	HB		x	
	MC	HB		x	
bD299	SC	HB		x	
bE302	SC	SB		x	
bR410	SC	HB	x		
bG424	MC	HB	x		
bG426	MC	HB	x		
bE436	SC	HB		x	
bK443	SC	HB	x		
bE439	SC	SB		x	

[a] MC, main chain; SC, side chain

[b] IA, interaction; HB, hydrogen bond; SB, salt bridge. Interactions were determined as defined in PDBePISA, version 1.48 (https://www.ebi.ac.uk/pdbe/pisa/).

[c] A distance cutoff of 3.5 Å was applied.

[d] The list also includes interactions with two structurally conserved water molecules that are coordinated by Mg^{2+} bound to the PabB synthase active site.

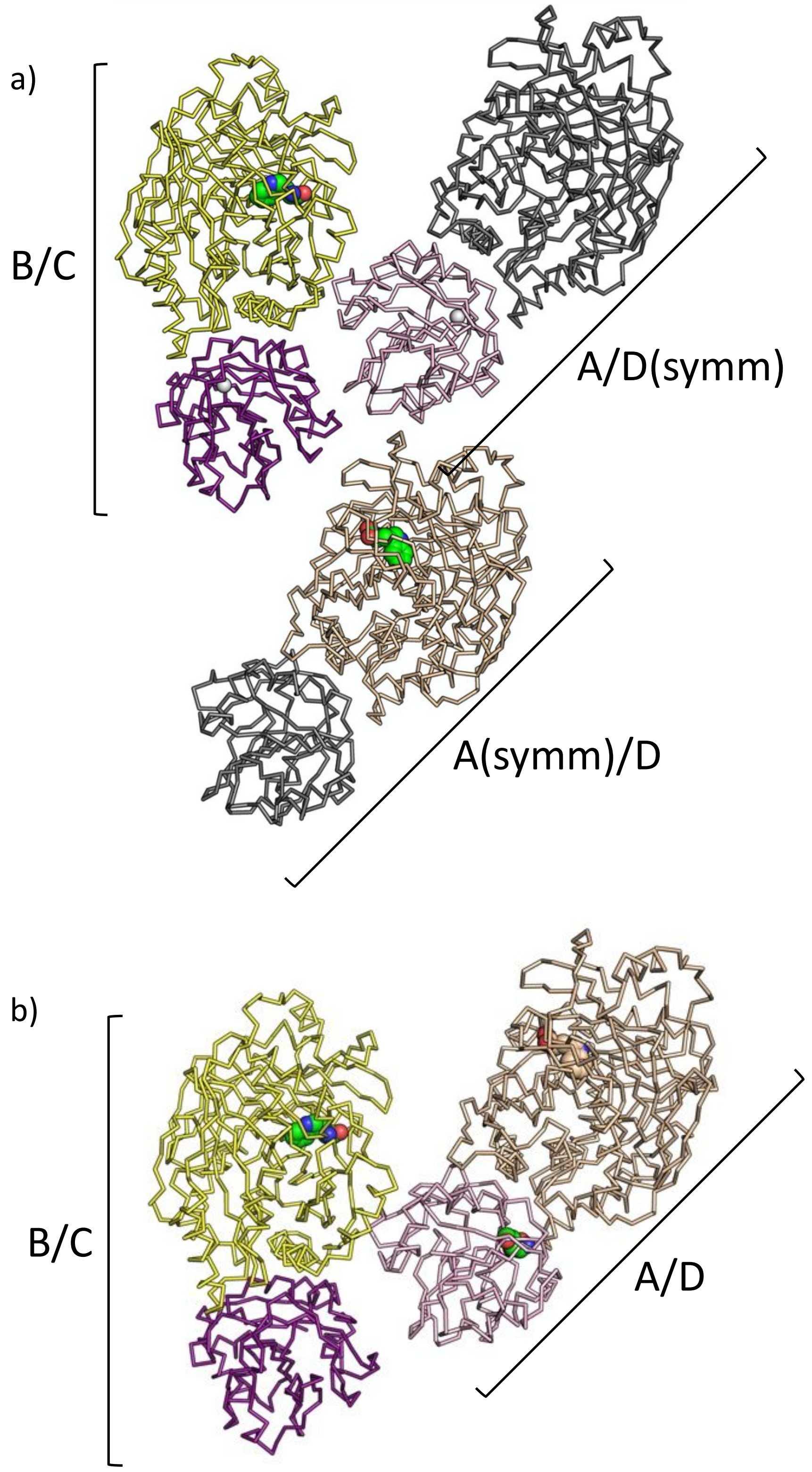

Supplementary Figure 1. ADCS complexes in different crystal packing arrangements. All crystal forms analyzed comprise two heterodimeric ADCS complexes **(*cf*. Supplementary Table 1). a,** in crystals used for ADCS structure determination without any reaction ligands or with added EDTA (PDB entries: 8RP6, 8RP2), the second ADCS complex is formed with symmetry-related subunits.

Two of these complexes are illustrated, in which the symmetry-related subunit is shown in grey, while the other one is shown in colors related to the first ADCS complex, using the color conventions from **Figure 2**; **b,** in crystals used for ADCS structure determination with Gln added to the crystallization buffer (PDB entries: 8RP1, 8RP0), both biologically relevant ADCS complexes are situated within the same asymmetric unit. The main difference between the two ADCS crystal forms is in a change of the c axis of approximately 5 Å (***cf*. Supplementary Table 1**). The color codes are as in panel a. Bound Trp is marked by sphere presentation to facilitate orientation. For structure-based PabA/PabB interface analysis, we used only those PabA/PabB heterodimeric assemblies formed within the same asymmetric unit, to exclude any uncertainty in the measured distances due to experimental errors in the unit cell calculation **(Supplementary Table 3**).

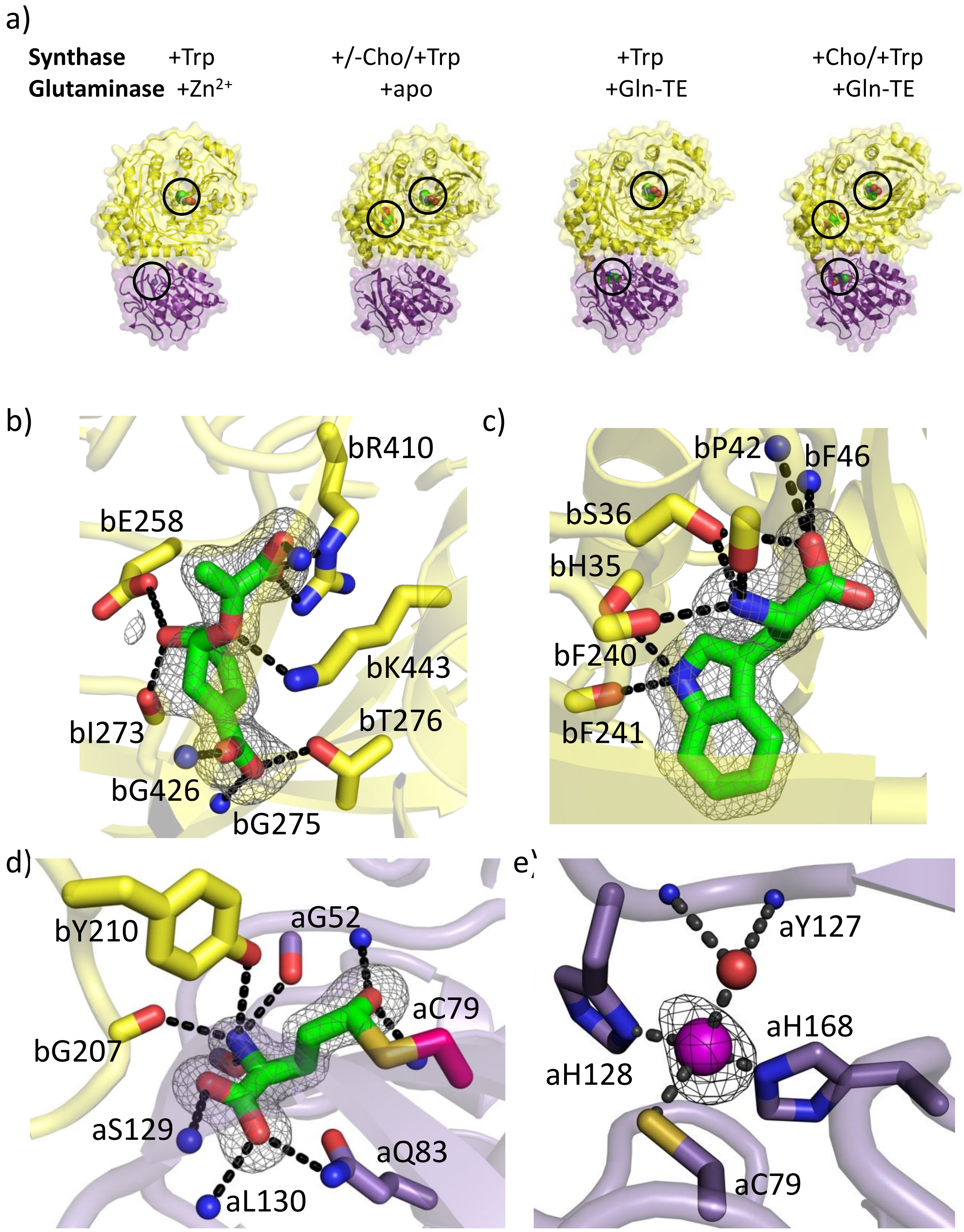

Supplementary Figure 2: ADCS ligand binding sites, as evidenced by *2Fo-Fc* omit density at about 1 σ level. a, Semi-transparent surface/cartoon representations of ADCS structures in different catalysis states, taken from **Figure 2b** as point of reference; **b**, Cho binding site (PDB entry: 8RP0, chain C) **(*cf*. Supplementary Figure 3, Supplementary Table 3); c**, Trp binding site (PDB entry: 8RP0,

chain D) (***cf*. Supplementary Table 3)**; d, Gln-TE binding site (PDB entry: 8RP0, chain A) (***cf*. Figures 3c, 4a, Supplementary Table 3)**; e, Zn^{2+} binding site (PDB entry: 8RP6 chain B) (***cf*. Figure 3a, Supplementary Table 3)**. Color codes are as in **Figure 3**. Selected interacting residues are shown in stick representation and are labeled. Hydrogen bonds are shown by dashed lines.

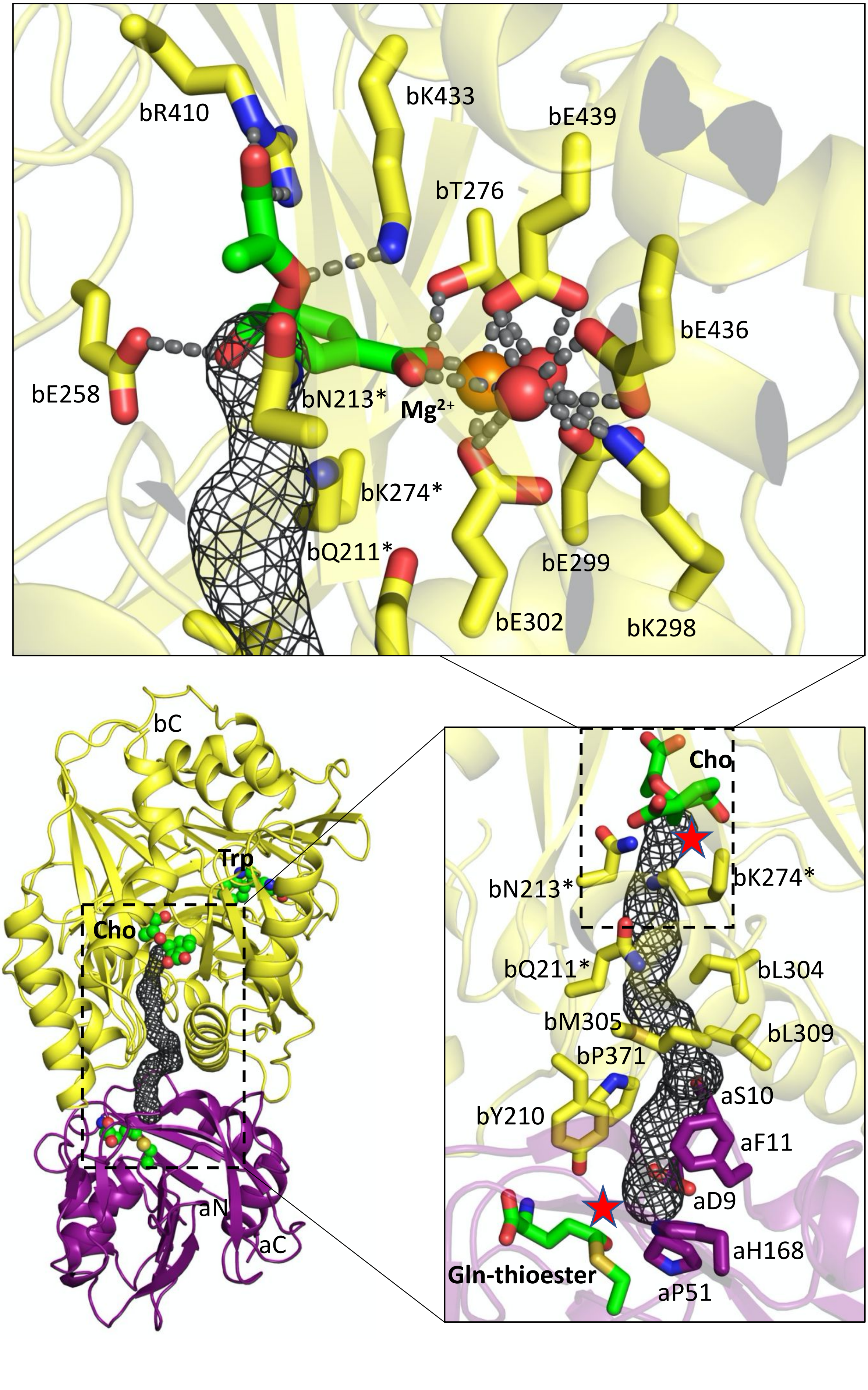

bR410
bK433
bE439
bT276
bE436
bE258
bN213*
Mg2+
bK274*
bQ211*
bE299
bE302
bK298
bC
Trp
Cho
aN
aC
Cho
bN213*
bK274*
bQ211*
bL304
bM305
bL309
bP371
aS10
bY210
aF11
aD9
aH168
Gln-thioester
aP51

116

Supplementary Figure 3: Structural details of ADCS glutaminase/synthase active sites-connecting ammonia tunnel. Cartoon representation (lower left), showing ADCS complex formation and glutaminase/synthase active site connecting ammonia tunnel. Zoom on the ADCS ammonia tunnel (lower right). Residues interacting with dummy atoms of the ammonia tunnel are shown and labelled. The approximate position of ammonia leaving from Gln upon Gln-TE formation is indicated by a red asterisk. Zoom on the Cho/Mg^{2+}-binding site (upper panel), which also includes two structurally conserved water molecules (red spheres), tightly bound to Mg^{2+} (orange sphere) **(Supplementary Table 7)**. The exit of the glutaminase/synthase active sites connecting the ammonia tunnel is indicated by a grey mesh. Residues that are shown in both zoom panels are indicated by asterisks. To enhance insight into the underlying structures, the orientation of ammonia tunnel and the Cho/Mg^{2+}-binding site zoom is different, corresponding to a rotation around a vertical axis of about 45 degrees. PDB entry 8RP0, chains A and D, was used to generate the Figure.

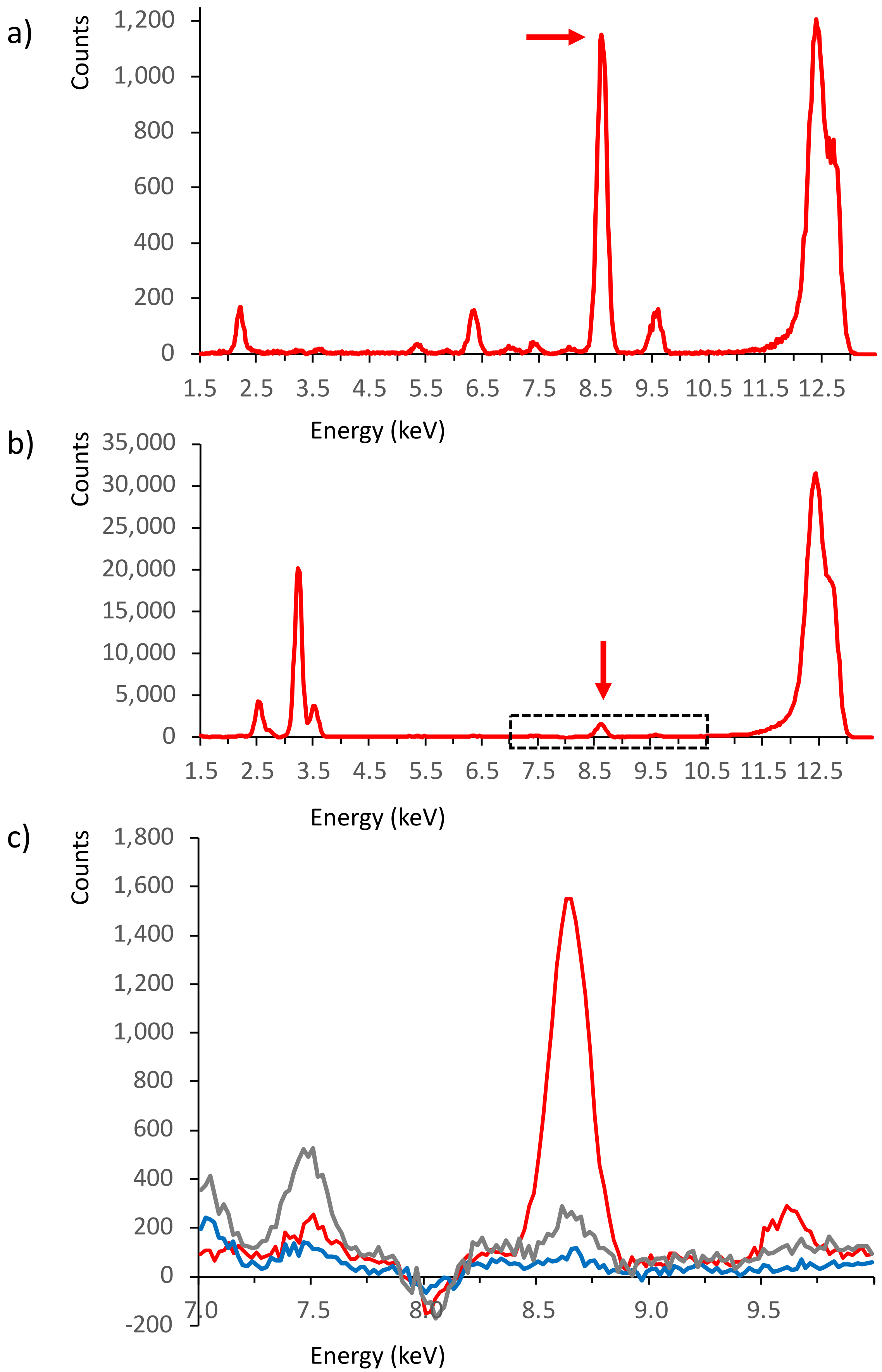
a)
Counts
1,200
1,000
800
600
400
200
0
1.5 2.5 3.5 4.5 5.5 6.5 7.5 8.5 9.5 10.5 11.5 12.5
Energy (keV)
b)
Counts
35,000
30,000
25,000
20,000
15,000
10,000
5,000
0
1.5 2.5 3.5 4.5 5.5 6.5 7.5 8.5 9.5 10.5 11.5 12.5
Energy (keV)
c)
Counts
1,800
1,600
1,400
1,200
1,000
800
600
400
200
0
-200
7.0 7.5 8.0 8.5 9.0 9.5
Energy (keV)

Supplementary Figure 4. Evidence for Zn^{2+} in the glutaminase active site. XRF scans of **a,** ADCS crystals without added reaction ligands; **b**, ADCS in solution without any reaction ligands added. Both spectra show a significant peak indicated by arrows at an energy that matches the $K_{\alpha 1}$ edge of zinc at 8.64 keV. The large peak at 12.4 keV represents the energy of the primary X-ray beam. The other large peak of ADCS in solution is due to the K_{α} edge of K^{+} at 3.31 keV from the buffer used for purified ADCS; **c**, Zoom into the 7.0-10.5 keV energy segment (boxed in panel b) of ADCS complexes in solution: untreated ADCS, red (identical to panel b); EDTA-treated ADCS, blue; untreated ADCS aH128A variant, grey.

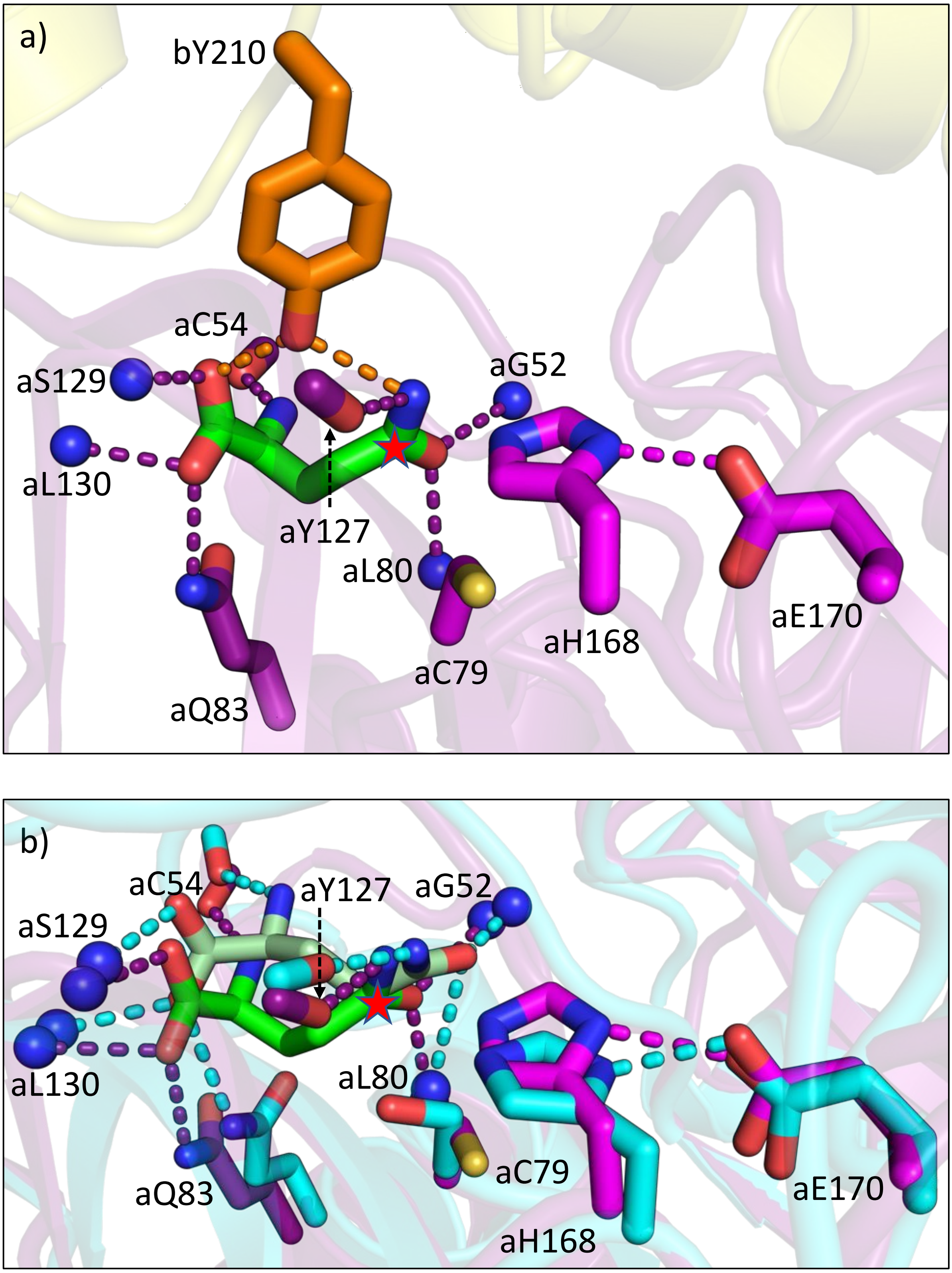

Supplementary Figure 5. Modeling of Gln in the ADCS glutaminase active site. a, Energy-minimized structural model of an ADCS Gln substrate complex (***cf*. Figure 3c**), based on the ADCS structure in the presence of Gln-TE **(Figure 4a, Supplementary Figure 2d)**; **b,** Superposition of the ADCS Gln

substrate complex with the Gln-bound glutaminase domain of carbamoyl phosphate synthetase (PDB code: 1C3O; active site residues in cyan, Gln in pale green, r.m.s.d. = 1.13 Å). Structurally conserved hydrogen bond interactions with glutaminase active site residues are indicated by dashed lines in matching colors, demonstrating the close resemblance of the model of Gln binding in the two systems. There are further GAT glutaminase structures with active site-bound Gln (PDB codes: 2NV2, 2F2A, 3ZR4, 7AC8), which are structurally more distantly related. The Gln-TE carbon atom, where the substitution of the substrate amino group leading to TE formation and subsequent Gln-TE hydrolysis takes place, is marked with a red asterisk.

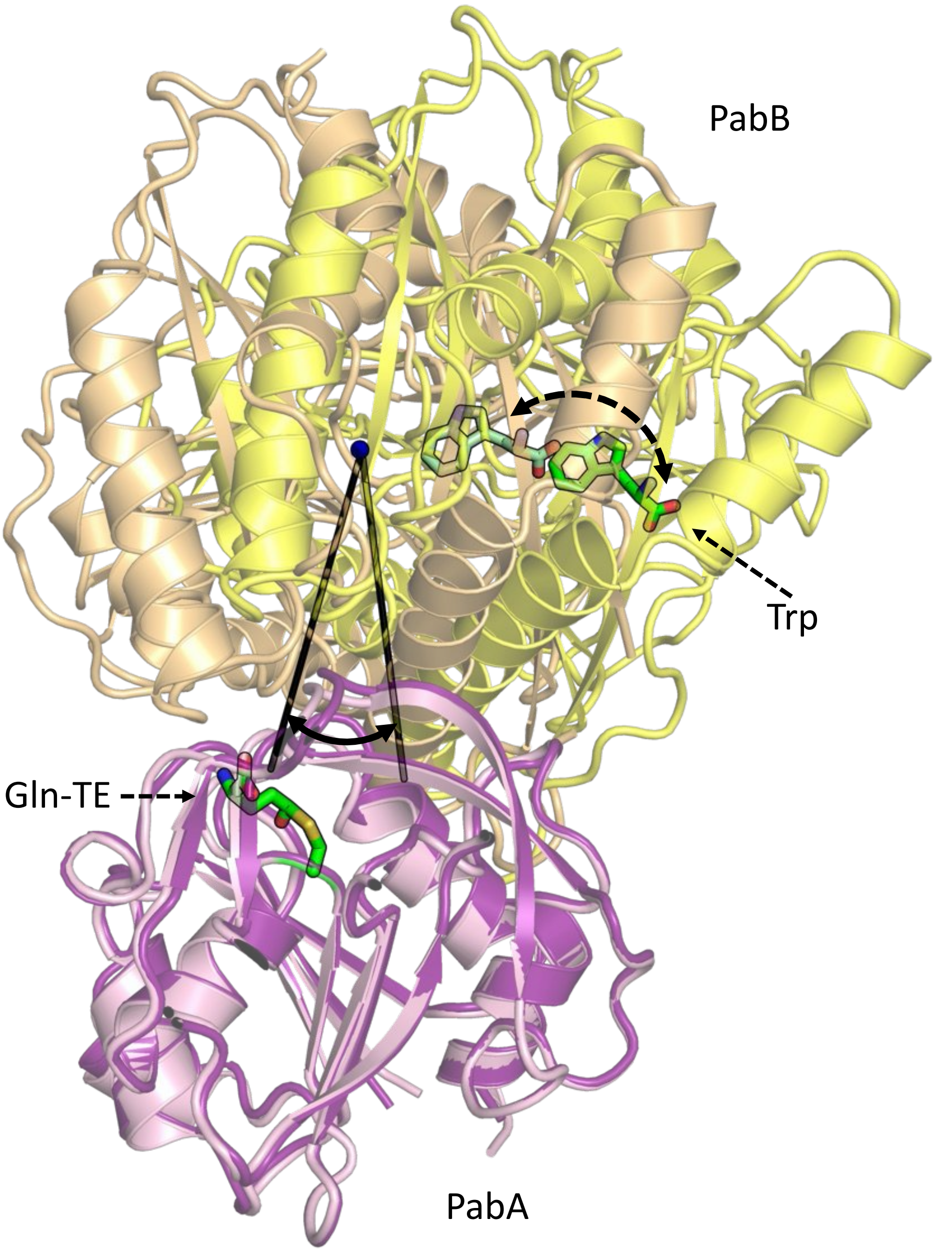

Supplementary Figure 6. Analysis of the rigid-body rotation between the PabA and PabB subunits of the ADCS complex upon Gln binding. PabA-based superposition of ADCS in the absence of reaction ligands (PDB entry: 8RP6 chains B and C) in colors as defined in **Figure 2** and Gln-TE bound ADCS (PDB entry: 8RP0 chains A and D) in related colors (PabA, light violet; PabB, light orange). The 23-degree rotation of the respective PabB subunits, as determined by PSICO/PyMol, is indicated. The Trp binding site in both structures is marked, to indicate the level of movement due to this rotational change. The bound Gln-TE in the glutaminase active site is also marked.

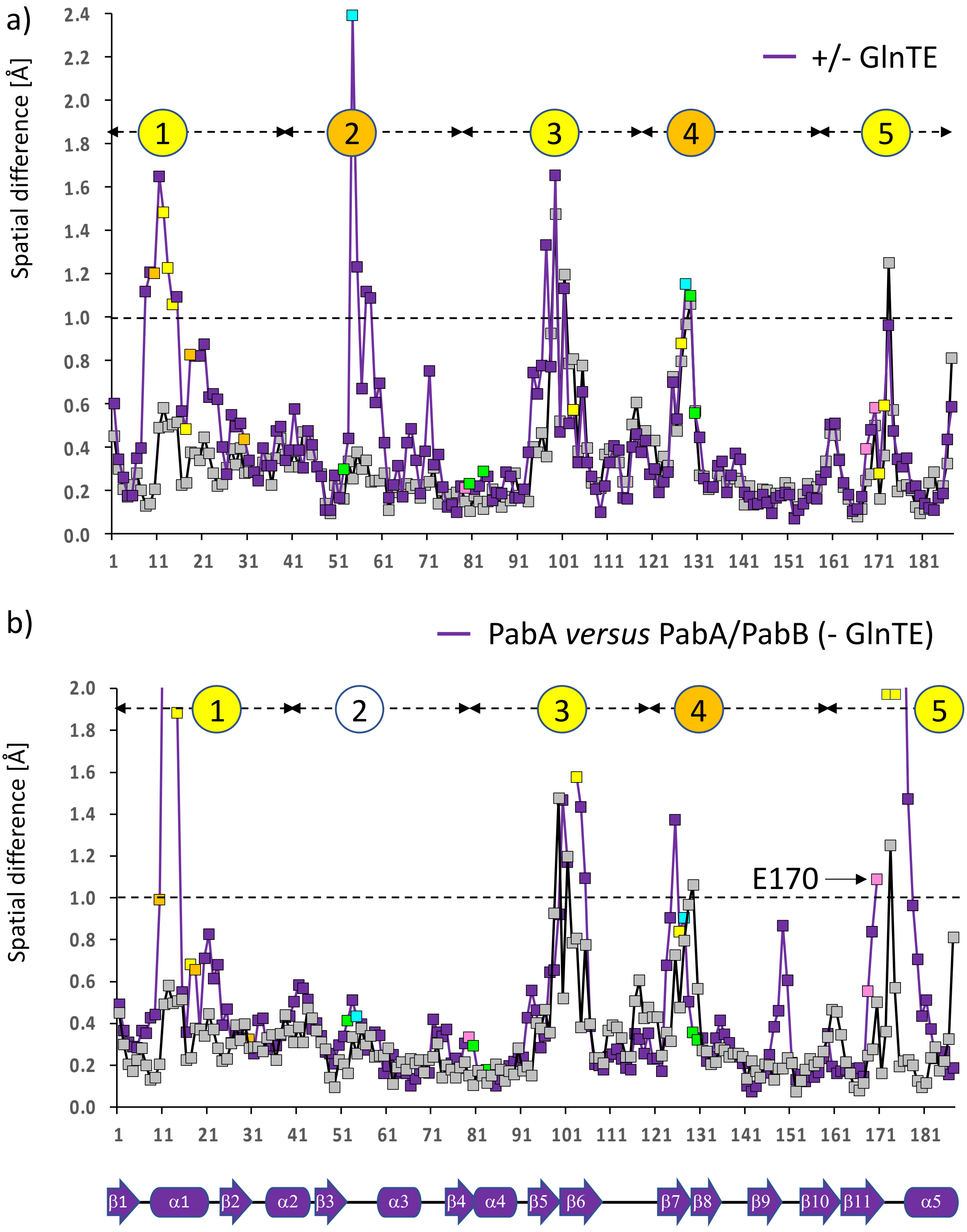

Supplementary Figure 7. PabA conformational changes induced by the presence of Gln-TE and PabB: a, R.m.s.d. plot indicating averaged spatial differences of PabA residues by superimposing structures of two ADCS heterodimeric complexes, where one PabA subunit is in the presence and the other one in the absence of Gln-TE (purple, PDB entries: 8RP1 chain A superimposed on 8RP6 chains A and B). **b,** R.m.s.d. plot indicating averaged spatial differences of PabA residues of the three

separate PabA molecules in purple (PDB entry: 8RP7). For comparison, averaged spatial differences of PabA residues are also shown in grey, by superimposing structures of two ADCS heterodimeric complexes, in which both PabA subunits are devoid of Gln-TE (PDB entries: 8RP1 chain B superimposed on 8RP6 chains A and B). Residues that are involved in different parts of the PabA/PabB interface are colored as in the previous figures**.** The plots indicate five highly flexible sequence segments with spatial differences exceeding 1 Å, indicated by a dashed line. These segments are numbered 1-5 and shown in colors, representing their roles in different parts of the PabA/PabB interface (*cf*. **Figure 5**). The sequence positions of the secondary structural elements found in the structures of PabA are indicated below in linear cartoon representation. In contrast, we did not detect any degree of conformational changes in any of the PabB segments involved in the PabA/PabB interface **(Supplementary Figure 11)**, demonstrating that the glutaminase active site rigidification due to ADCS assembly is restricted to PabA.

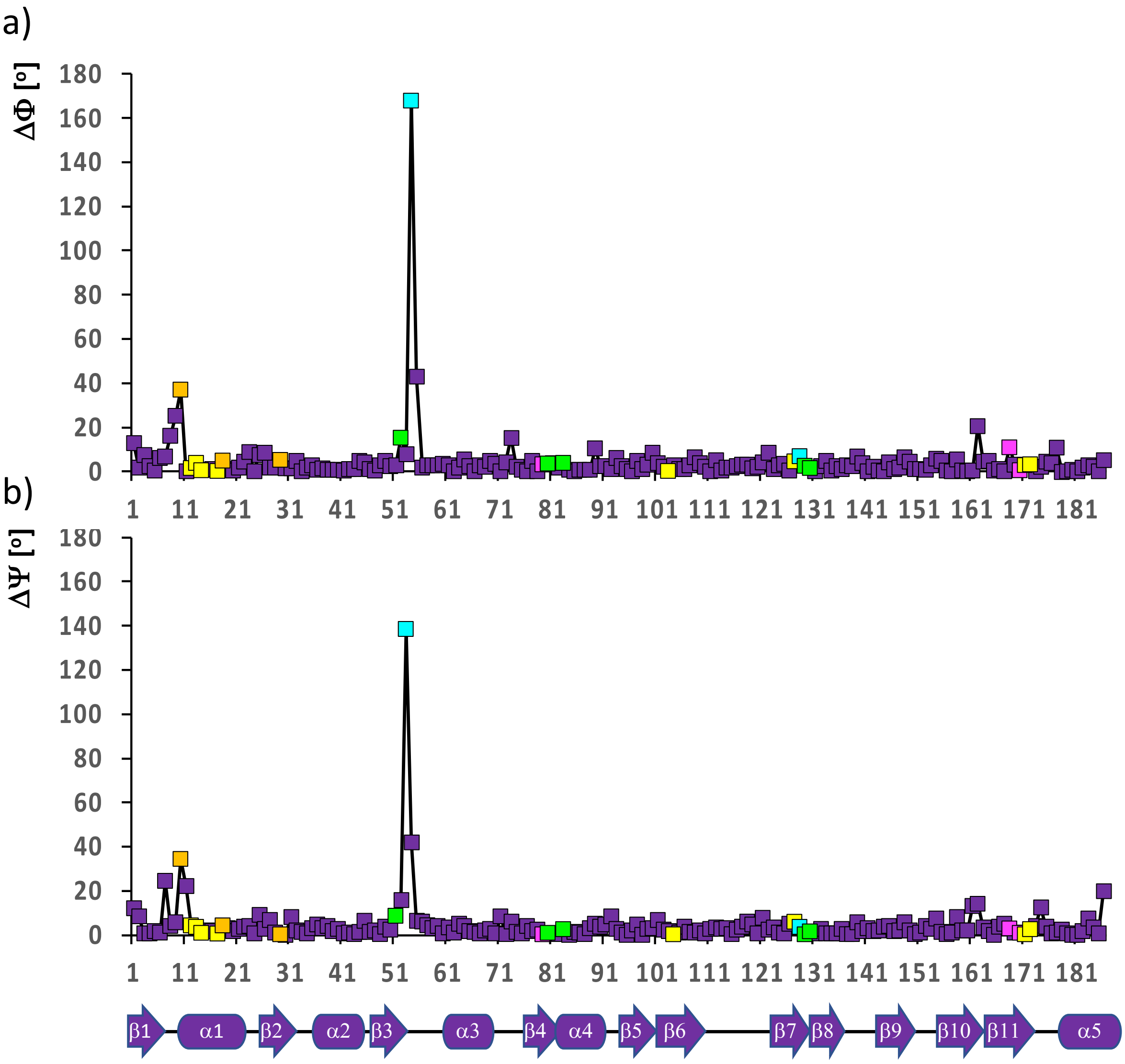

Supplementary Figure 8. PabA dihedral angle changes induced by the presence of Gln-TE. a, Absolute values of dihedral Φ angle differences of PabA subunits in the presence and absence of Gln-TE; **b,** absolute values of dihedral Ψ angle differences of PabA subunits in the presence and absence of Gln-TE.

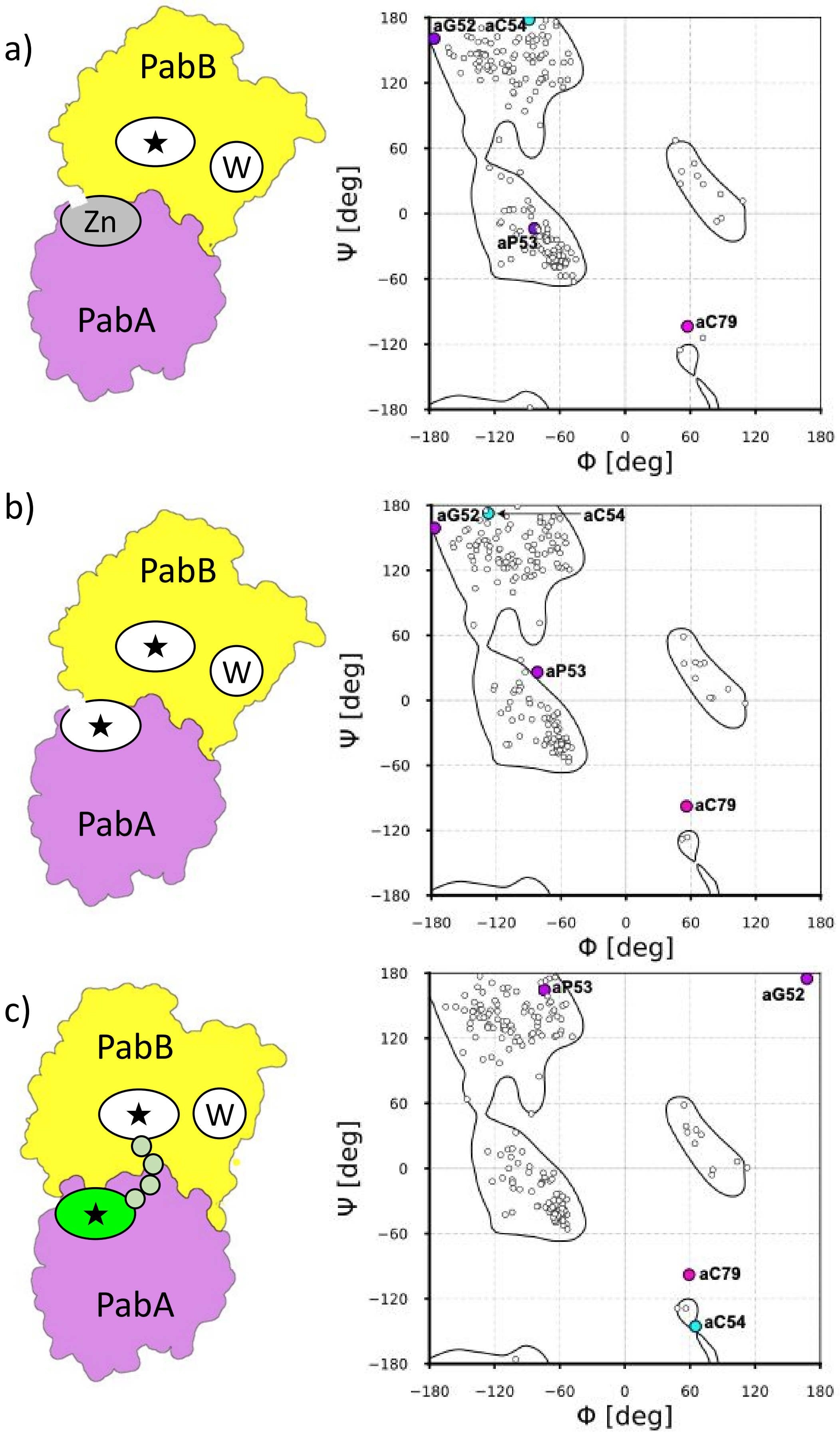
a)
PabB
W
Zn
PabA
aG52
aC54
aP53
aC79
Ψ [deg]
Φ [deg]
b)
PabB
W
PabA
aG52
aC54
aP53
aC79
Ψ [deg]
Φ [deg]
c)
PabB
W
PabA
aP53
aG52
aC79
aC54
Ψ [deg]
Φ [deg]

Supplementary Figure 9. Ramachandran plot of PabA. a, Plot in presence of Zn^{2+} (PDB code: 8RP6 chain B), **b,** in absence of any glutaminase active site ligands (PDB code: 8RP2 chain B) or **c**, with bound Gln-TE (PDB code: 8RP0 chain A). Residues aG52, aP53, aC54 and aC79 are highlighted. Color codes are as in **Figure 3.** Corresponding schematic ADCS PabA/PabB arrangements are shown on the left (***cf*. Figure 2**).

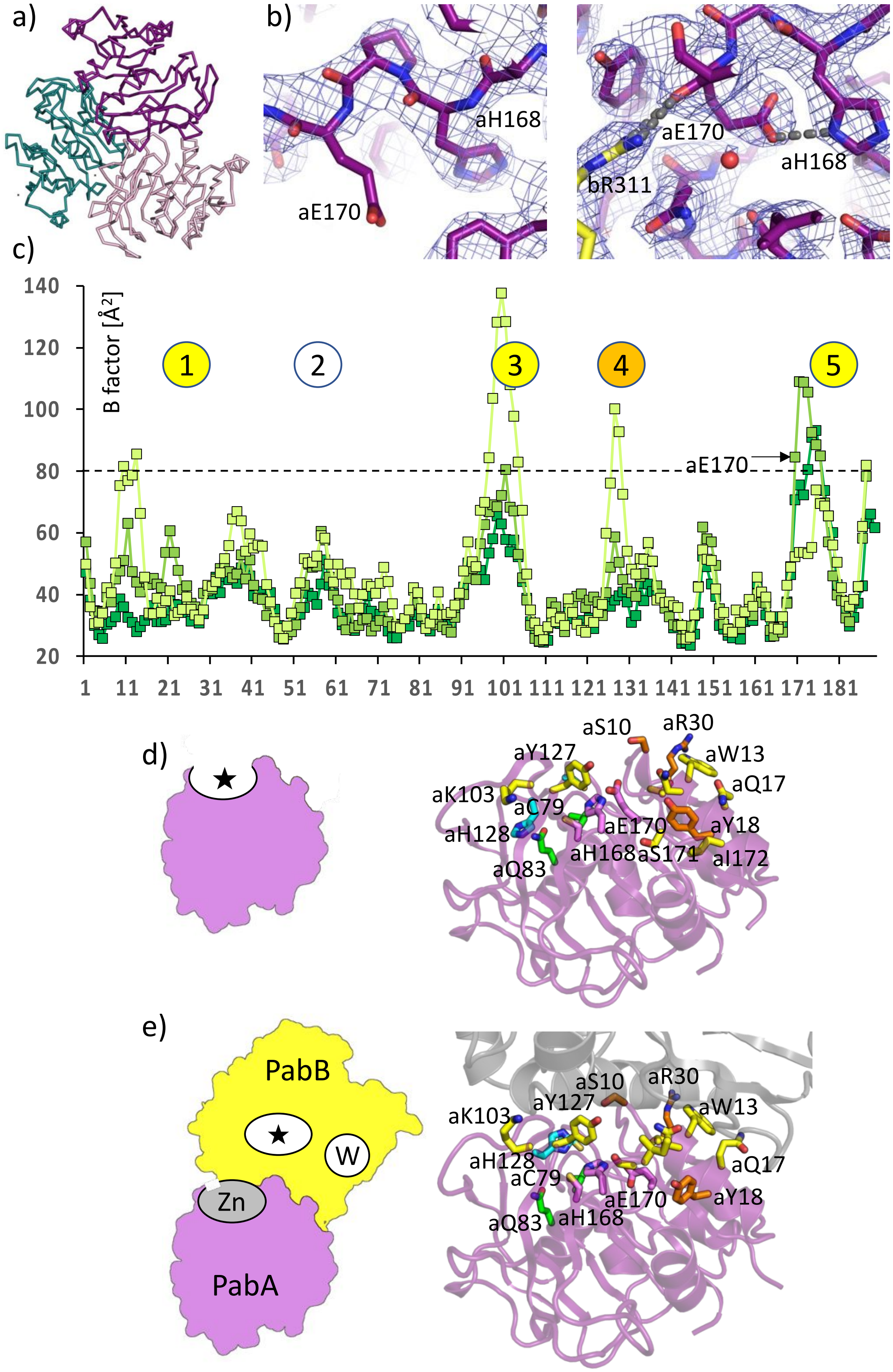
a)
b)
aH168
aE170
aE170
aH168
bR311
c)
B factor [Å2]
140
120
100
80
60
40
20
1
2
3
4
5
aE170
1 11 21 31 41 51 61 71 81 91 101 111 121 131 141 151 161 171 181
d)
aS10
aR30
aY127
aW13
aK103
aQ17
aC79
aE170
aH128
aY18
aH168
aS171
aI172
aQ83
e)
PabB
W
Zn
PabA
aS10
aR30
aY127
aW13
aK103
aH128
aQ17
aC79
aE170
aY18
aQ83
aH168

Supplementary Figure 10. Increased conformational flexibility of the glutaminase active site in separate PabA subunits. a, Arrangement of three separate PabA subunits within the crystal lattice (PDB entry: 8RP7). **b**, Left panel: Loose arrangement of catalytic triad residues aH168 and aE170 in separate PabA (PDB entry: 8RP7, chain A). Right panel: Catalytically competent arrangement of catalytic triad residues aH168 and aE170 in ADCS complexes (PDB entry: 8RP2, chains B and C), for comparison **(*cf*. Figures 3d, 4a)**. Hydrogen bonds connecting the side chains of aH168 and aE170, and the main chain carbonyl group of aE170 with the side chain of bE311 from the PabB subunit, are shown by dashed lines in grey. The structural models are displayed within the corresponding *2Fo-Fc* density maps used for modeling, to highlight the observed rigidification of the catalytic triad residues by the presence of PabB. In separate PabA, the high flexibility of the glutaminase active site leads to variable catalytic triad (aC79, aH68, aE170) arrangements, in which especially aE170 does not consistently adopt a position that allows to play its functional role as a relay of aH168 to enhance its function as nucleophile during glutaminase catalysis **(Figures 3c-d, 4a).** In contrast, in the structures of ADCS complexes there is a specific interaction between the main chain carbonyl group of aE170 and bR311, indicating an additional crucial role of PabB to generate a catalytically competent conformation of the glutaminase catalytic triad, irrespective of the presence of Gln **(Figure 5). c,** Residual mobility plots derived from refined crystal structures of the three separate PabA molecules (PDB entry: 8RP7) in different green shades. High mobility areas are numbered and match the structurally most divergent regions in PabA **(Supplementary Figure 7).** Cartoon representation of **d,** the separate PabA subunit structure (PDB entry: 8RP7, chain A) and **e,** the PabA subunit of the ADCS complex in complex with PabB, in the absence of Gln-TE (PDB entry: 8RP6). Color codes: PabA, violet; PabB, grey. PabA residues involved in the PabA/PabB interface in ADCS complex structures are shown in complementary PabB residue colors **(*cf*. Figure 5c, d).** Corresponding schematic PabA and PabA/PabB ADCS complex arrangements **(*cf*. Figure 2c)** are shown on the left.

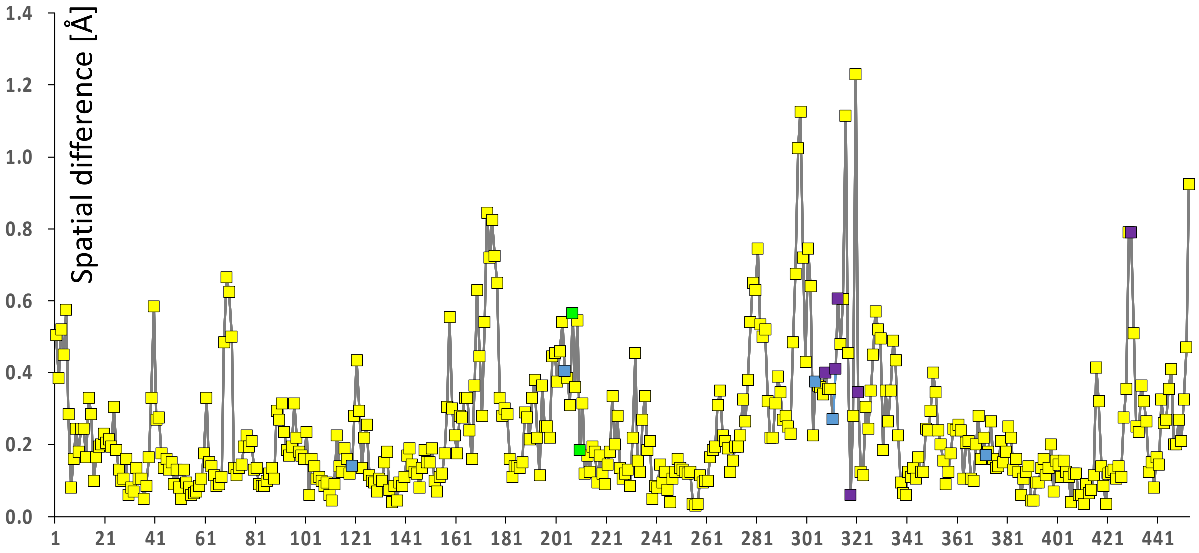

Supplementary Figure 11. **The PabB mobility is not significantly affected by Gln-TE binding to the ADCS complex.** R.m.s.d. plot indicating averaged spatial differences of PabB residues by superimposing structures of two heterodimeric ADCS complexes, where one interacting PabA subunit is in the presence and the other one in the absence of Gln-TE (PDB entries: 8RP1 chain D superimposed on 8RP6 chains C and D). A similar plot for the PabA sequence is shown in **Supplementary Figure 7a.** Note that most PabA/PabB interface residues from the PabB sequence do not reveal large scale spatial deviations, contrary to observations for PabA**.** Colors deviating from yellow are used for PabB residues contributing to the generic PabA/PabB interface in dark violet, contributing to the Gln-TE induced extended PabA/PabB interface in blue, and directly interacting with Gln-TE (when present) in green.

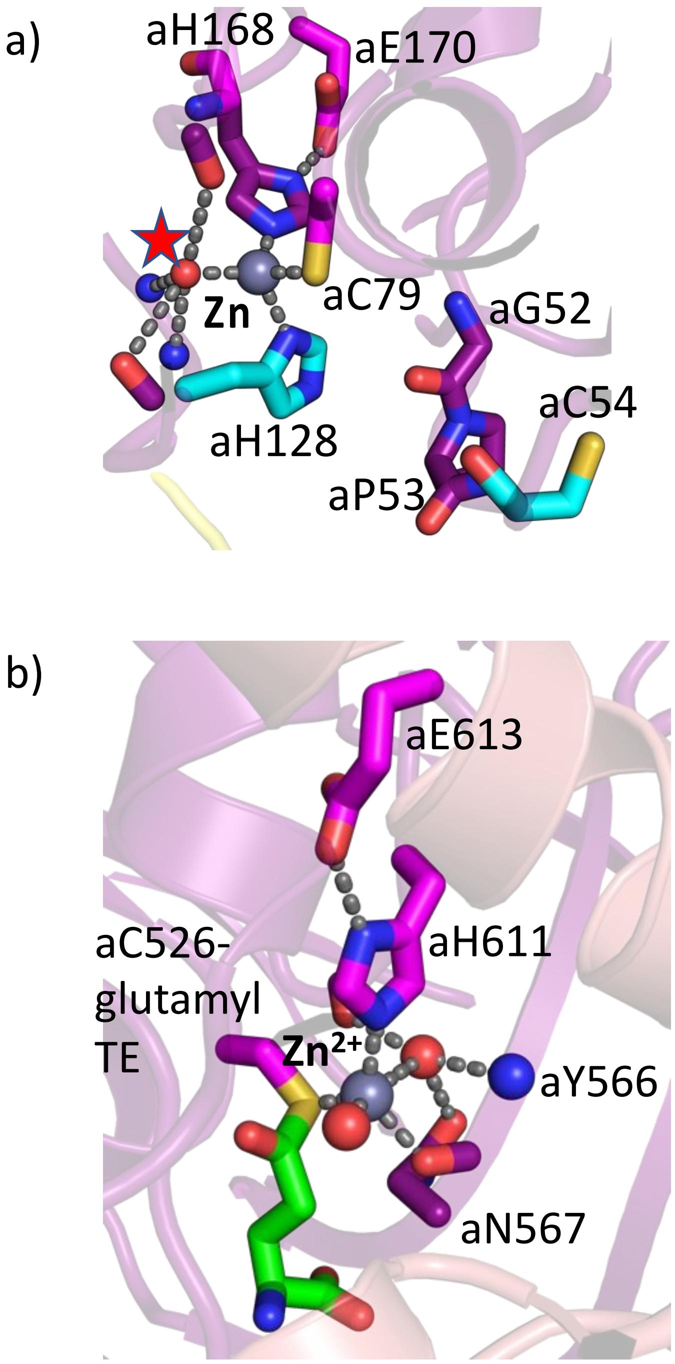

Supplementary Figure 12. **Structurally conserved Zn^{2+} in the glutaminase active sites** of **a**) ADCS (***cf*. Figure 3a)** and **b**) ADICS (PDB code: 3R75). Contrary to ADICS, in which Zn^{2+} binding was observed in the presence of Gln-TE and could potentially act as a Lewis acid to enhance catalysis [2], in ADCS we found Zn^{2+} only in the absence of Gln-TE, coupled with a swinging motion of aH128 and essentially blocking the ADCS glutaminase active site for substrate access **(Figure 3).** In ADICS, no comparable conformational change of any residue next to the glutaminase active site upon Zn^{2+} binding was observed. Interestingly, in both GATs Zn^{2+} appears to be extracted from the expression host, as no

zinc ions were added during the purification and crystallization procedure [3]. In both systems, zinc disappears from the glutaminase active site upon EDTA treatment, which is associated with no or only minor changes in glutaminase turnover **(Supplementary Table 4)** [3].

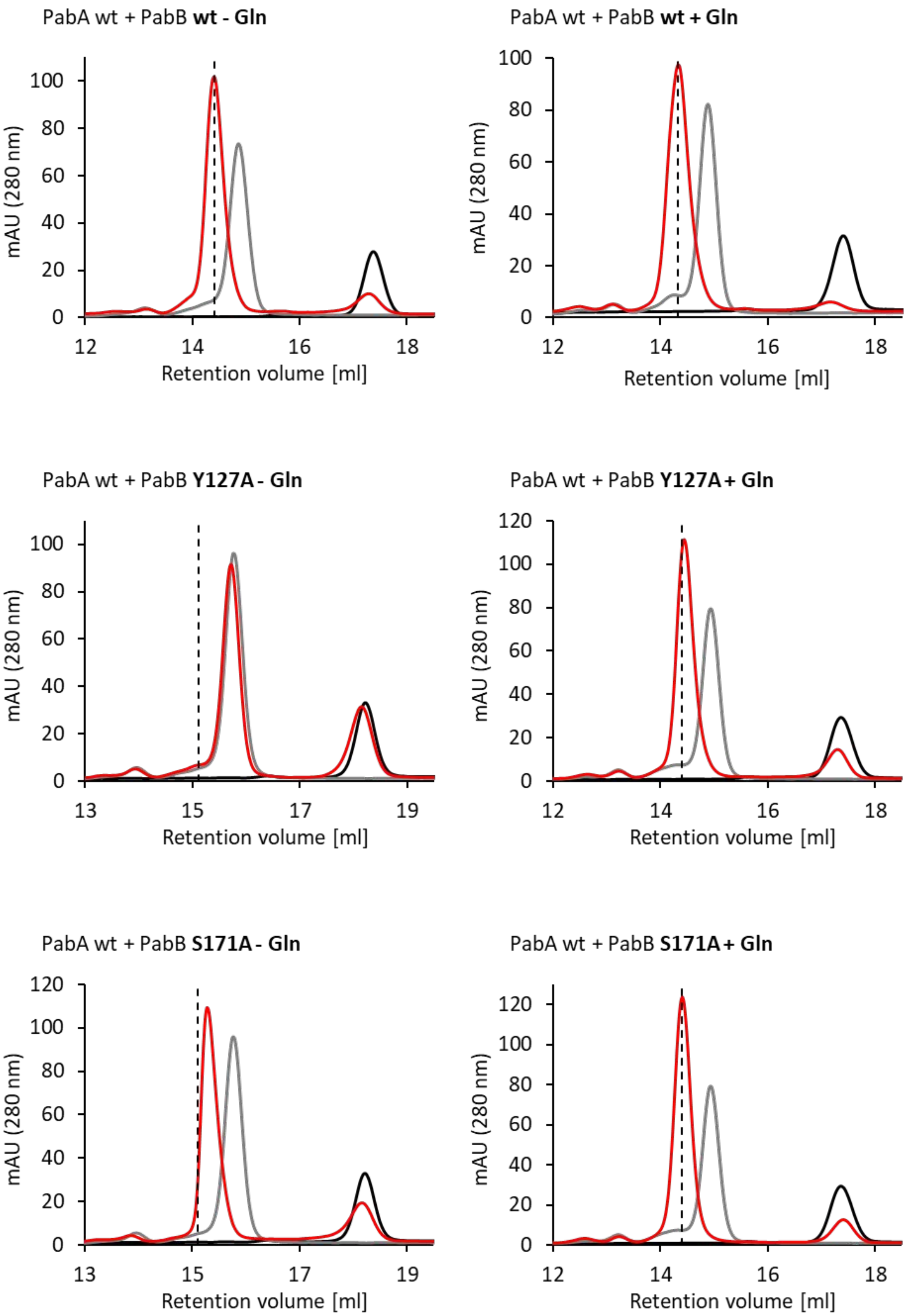
PabA wt + PabB wt - Gln
PabA wt + PabB wt + Gln
PabA wt + PabB Y127A - Gln
PabA wt + PabB Y127A + Gln
PabA wt + PabB S171A - Gln
PabA wt + PabB S171A + Gln
mAU (280 nm)
Retention volume [ml]
0
20
40
60
80
100
120
12
13
14
15
16
17
18
19

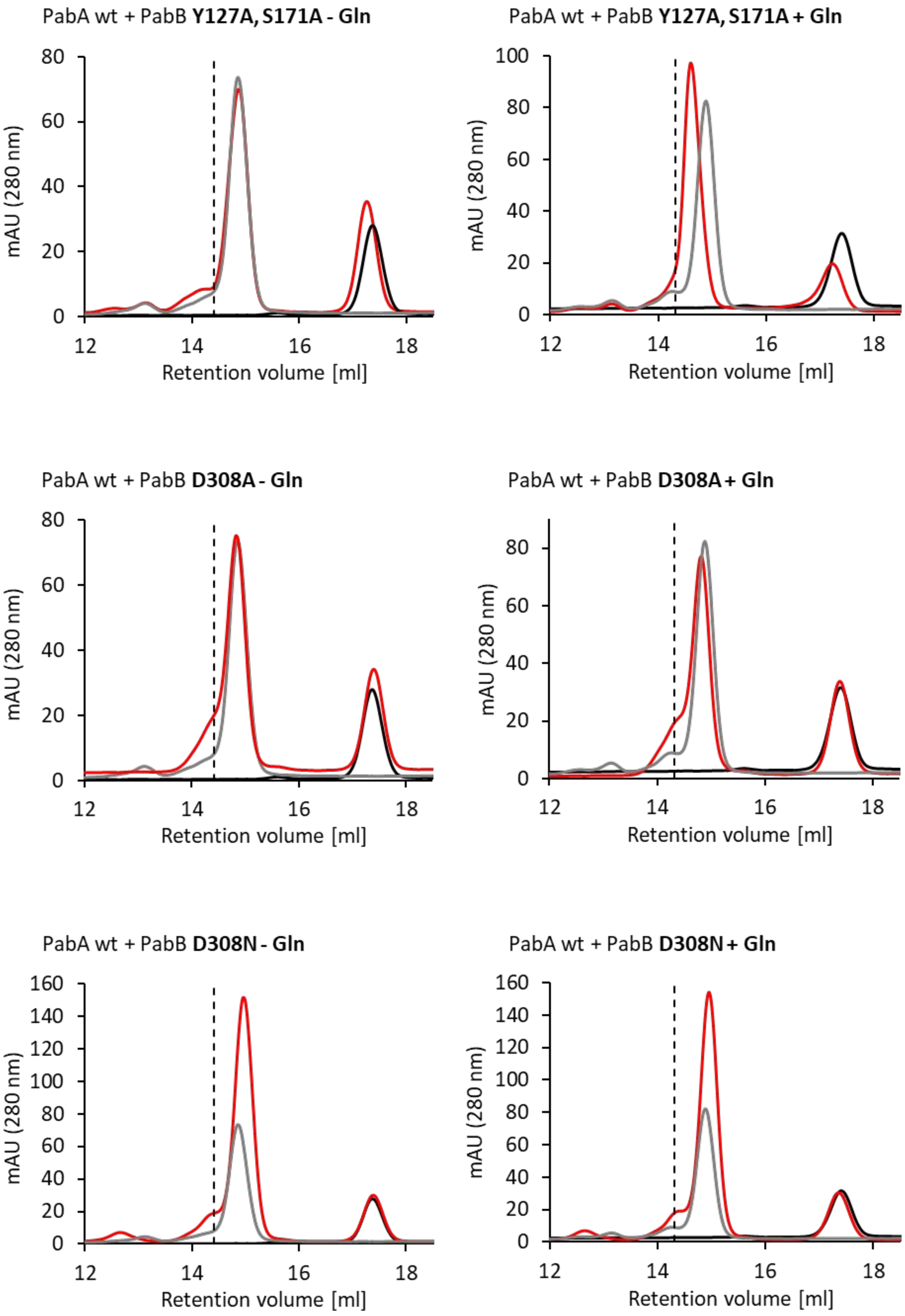
PabA wt + PabB **Y127A, S171A - Gln**
PabA wt + PabB **Y127A, S171A + Gln**
PabA wt + PabB **D308A - Gln**
PabA wt + PabB **D308A + Gln**
PabA wt + PabB **D308N - Gln**
PabA wt + PabB **D308N + Gln**
mAU (280 nm)
Retention volume [ml]
0
20
40
60
80
100
120
140
160
12
14
16
18

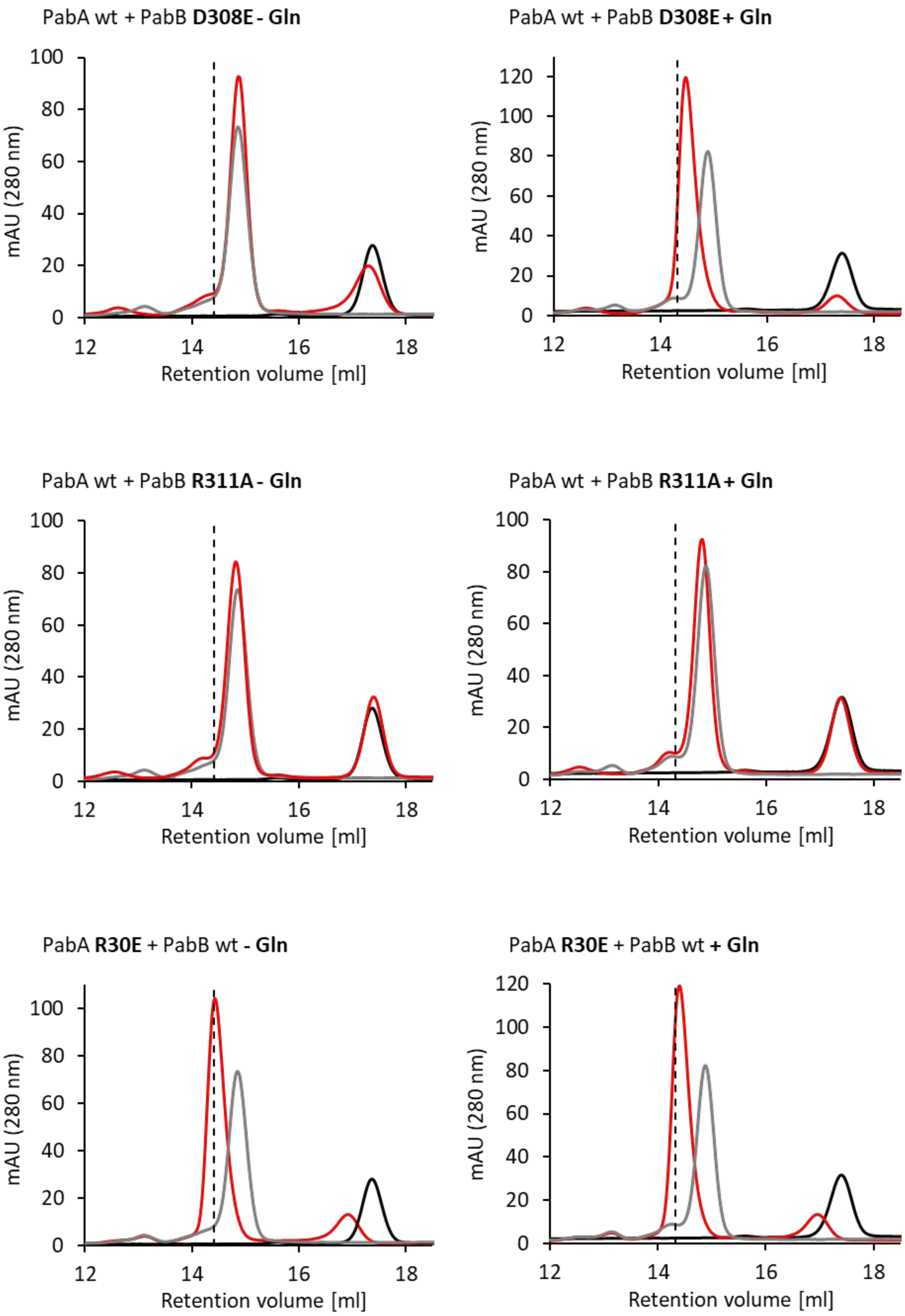
PabA wt + PabB **D308E - Gln**
PabA wt + PabB **D308E + Gln**
PabA wt + PabB **R311A - Gln**
PabA wt + PabB **R311A + Gln**
PabA **R30E** + PabB wt **- Gln**
PabA **R30E** + PabB wt **+ Gln**
mAU (280 nm)
Retention volume [ml]
0
20
40
60
80
100
120
12
14
16
18

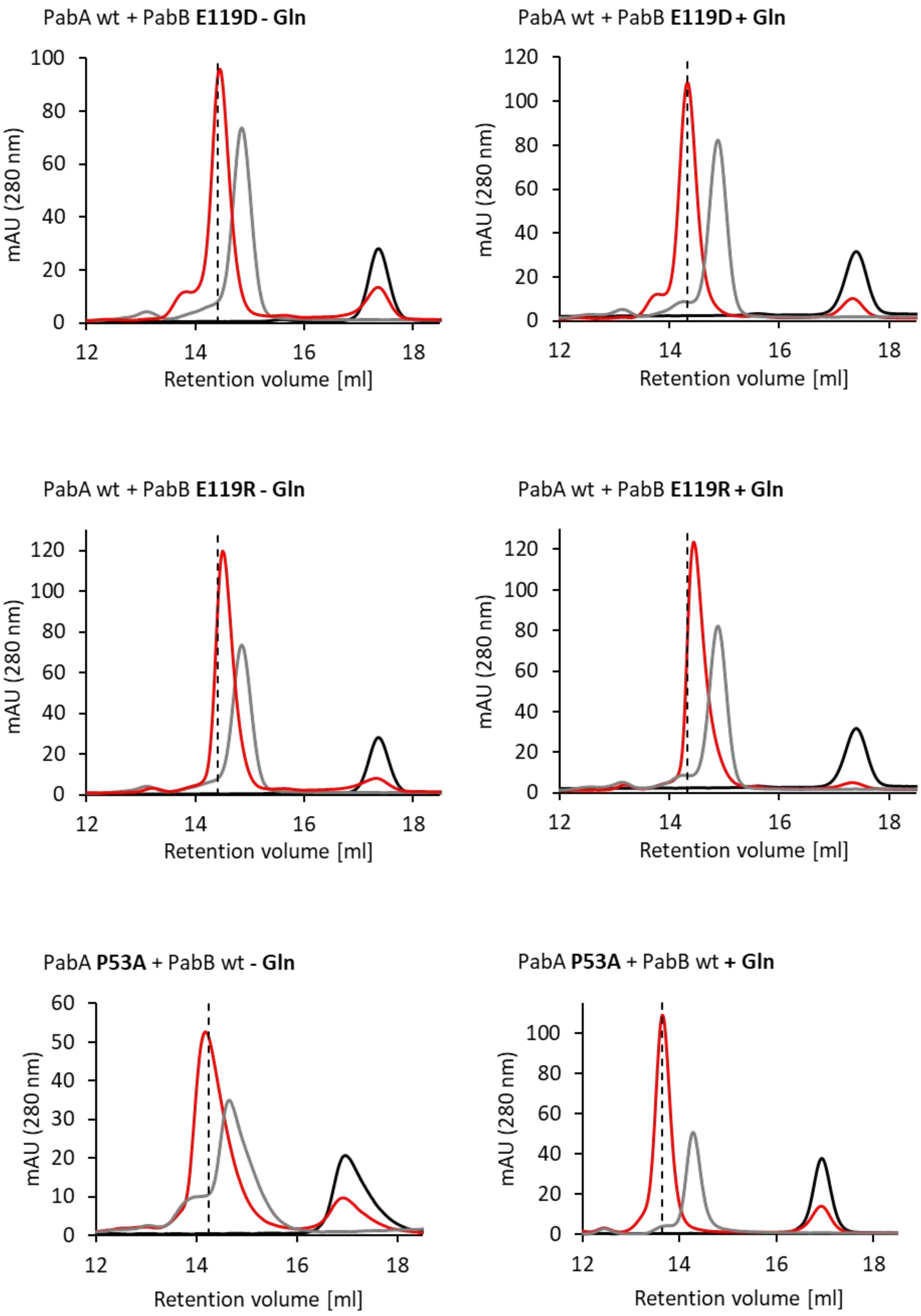
PabA wt + PabB **E119D - Gln**
PabA wt + PabB **E119D + Gln**
PabA wt + PabB **E119R - Gln**
PabA wt + PabB **E119R + Gln**
PabA **P53A** + PabB wt **- Gln**
PabA **P53A** + PabB wt **+ Gln**
mAU (280 nm)
Retention volume [ml]

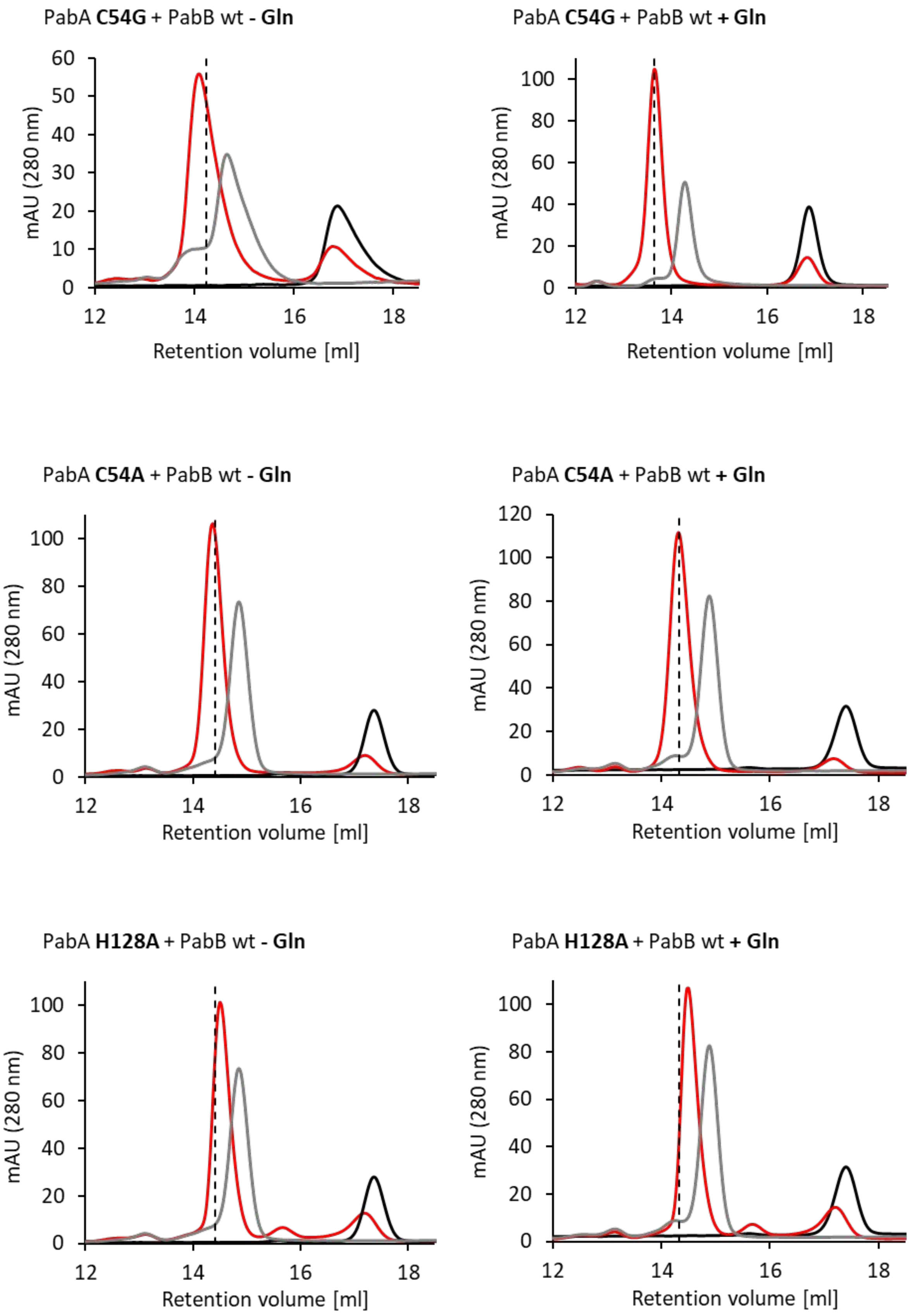
PabA **C54G** + PabB wt - **Gln**
PabA **C54G** + PabB wt **+ Gln**
PabA **C54A** + PabB wt - **Gln**
PabA **C54A** + PabB wt **+ Gln**
PabA **H128A** + PabB wt - **Gln**
PabA **H128A** + PabB wt **+ Gln**
mAU (280 nm)
Retention volume [ml]

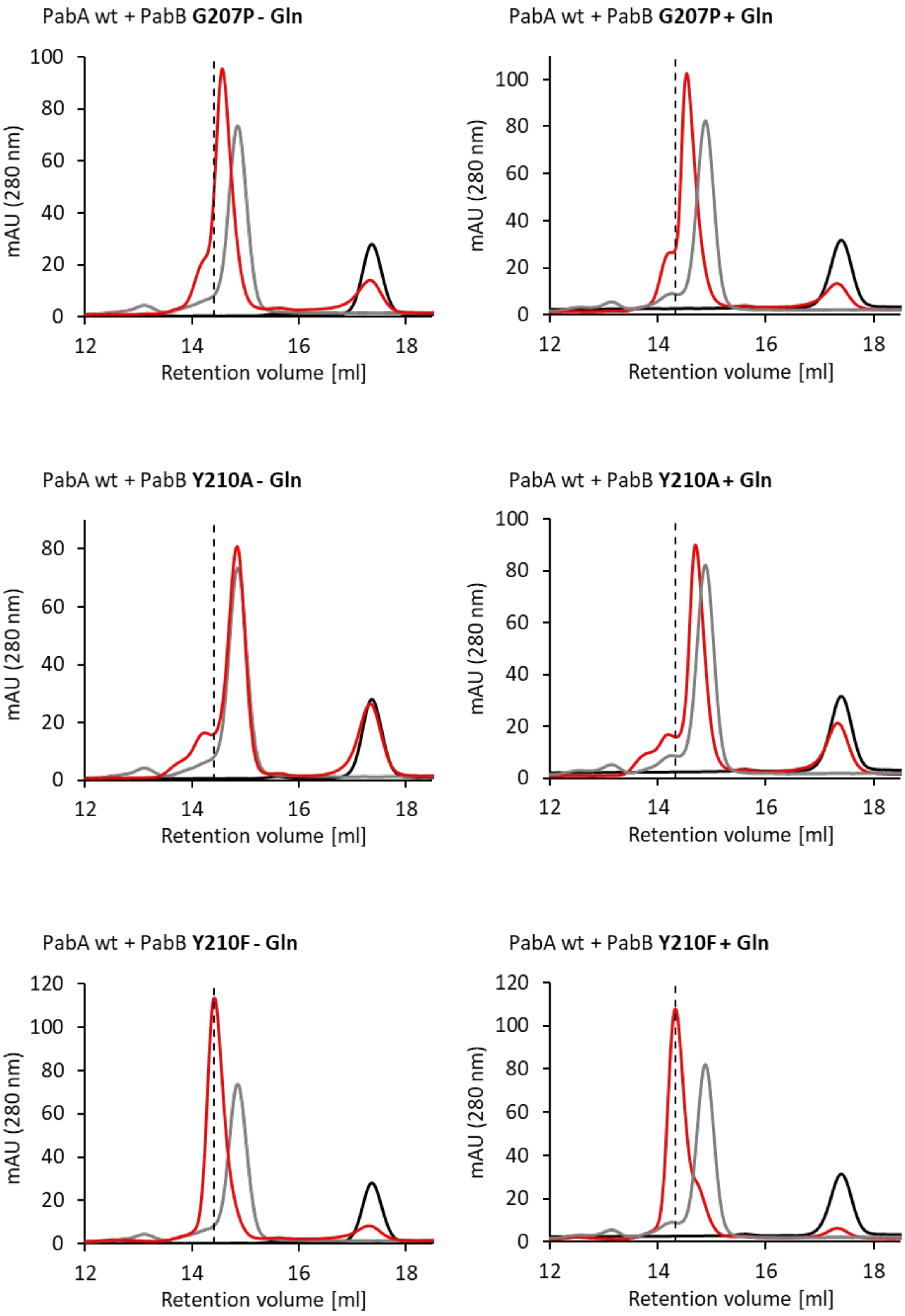
PabA wt + PabB **G207P - Gln**
PabA wt + PabB **G207P + Gln**
PabA wt + PabB **Y210A - Gln**
PabA wt + PabB **Y210A + Gln**
PabA wt + PabB **Y210F - Gln**
PabA wt + PabB **Y210F + Gln**
mAU (280 nm)
Retention volume [ml]
0
20
40
60
80
100
120
12
14
16
18

Supplementary Figure 13. Elution profiles of analytical SEC to analyze Gln-dependent PabA/PabB complex formation. Analytical SEC was performed on a Superdex 200 column equilibrated with 50 mM Tris/HCl pH 7.5, 50 mM KCl, 5 mM $MgCl_2$, 2 mM DTT and, where indicated, 5 mM Gln. Dashed line, retention volume of the *wt* PabA/PabB complex; black, *wt* PabA; grey, *wt* PabB; red, ADCS variant as indicated above each graph.

PabA wt + PabB wt

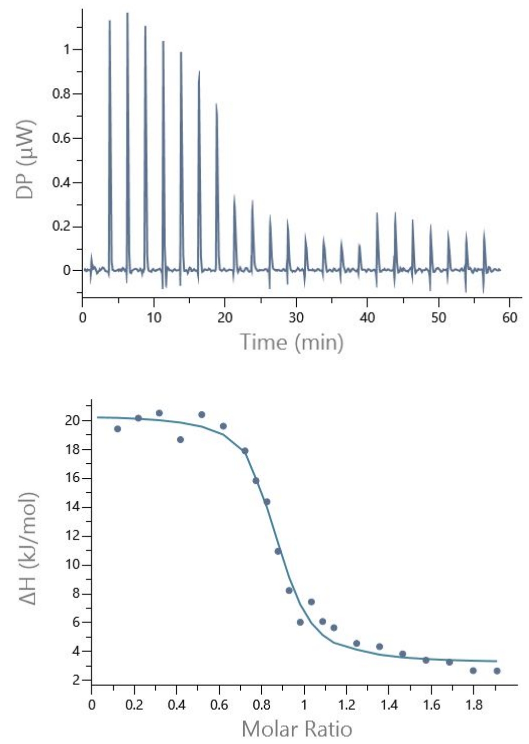

PabA Y127A + PabB wt

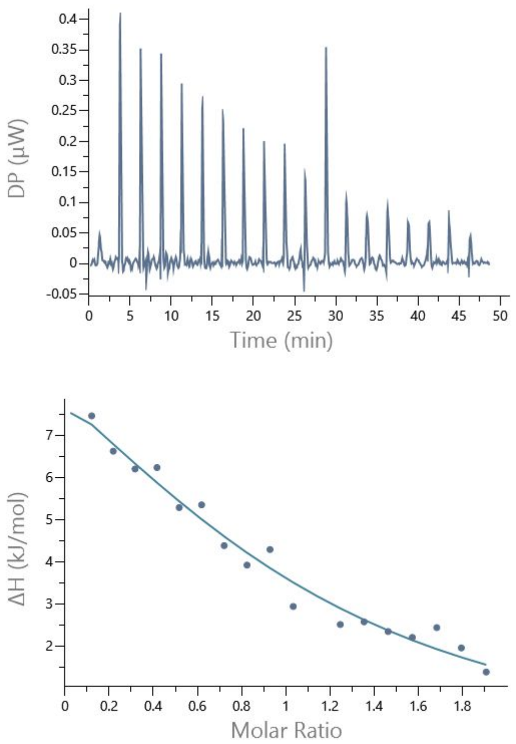

PabA S171A + PabB wt

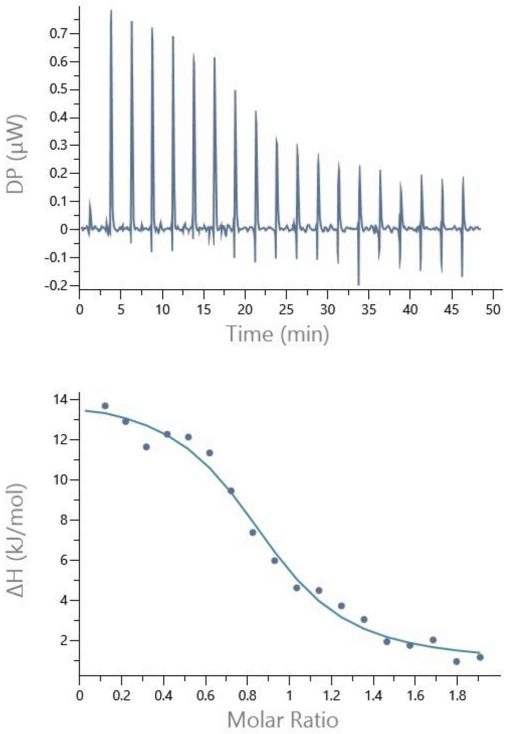

PabA [Y127A, S171A] + PabB wt

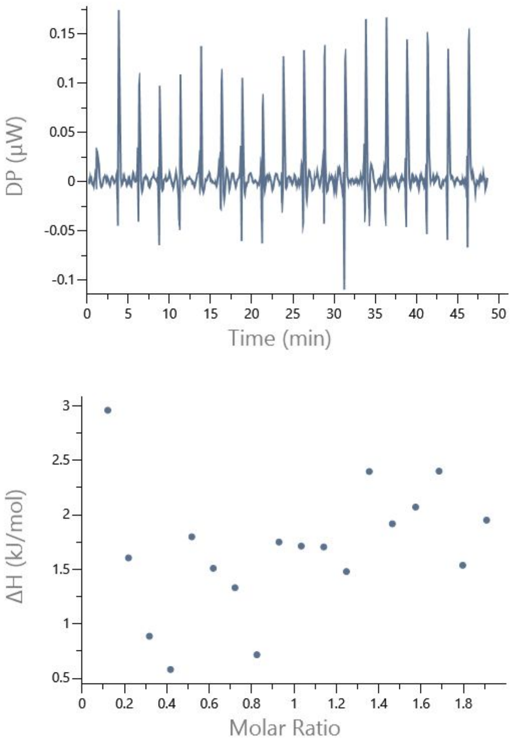

PabA wt + PabB D308A

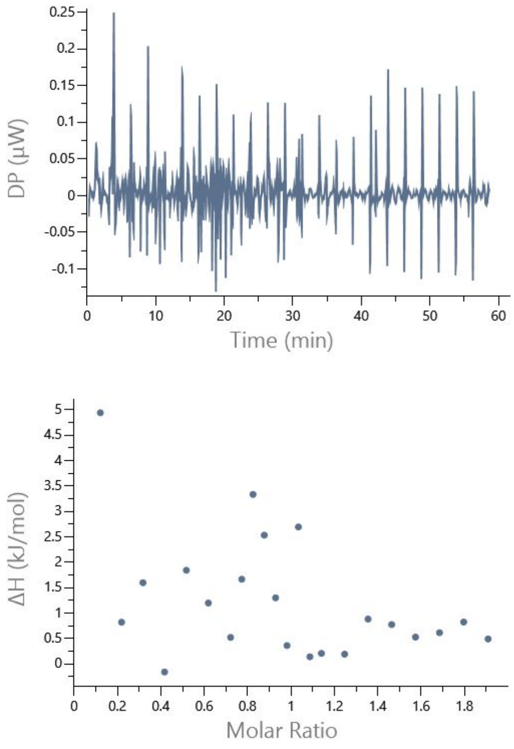

PabA wt + PabB D308N

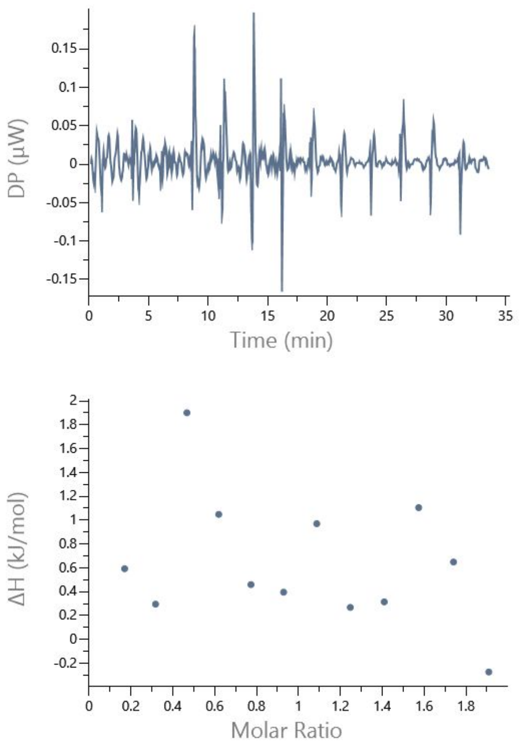

PabA wt + PabB D308E

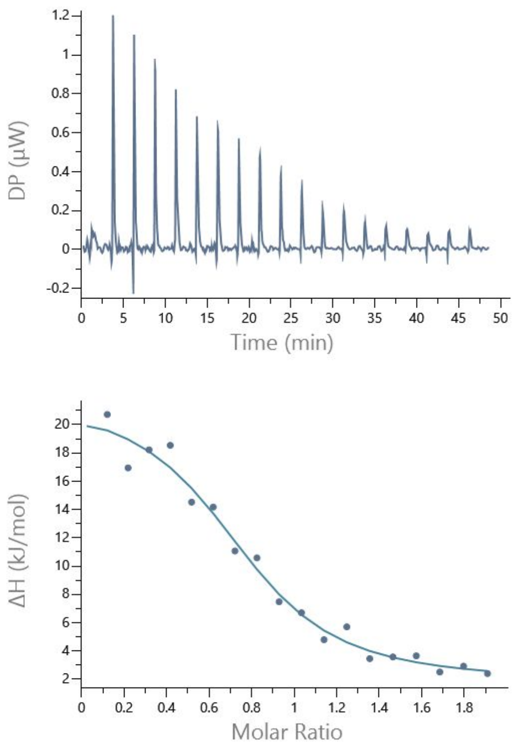

PabA wt + PabB R311A

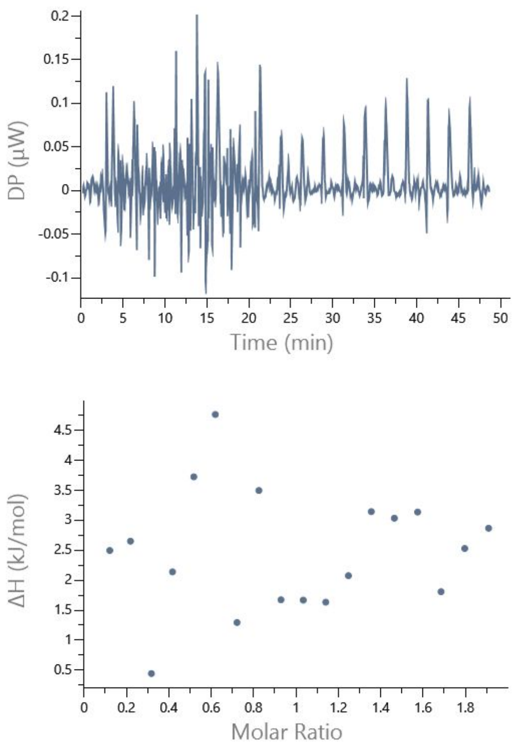

PabA R30E + PabB wt

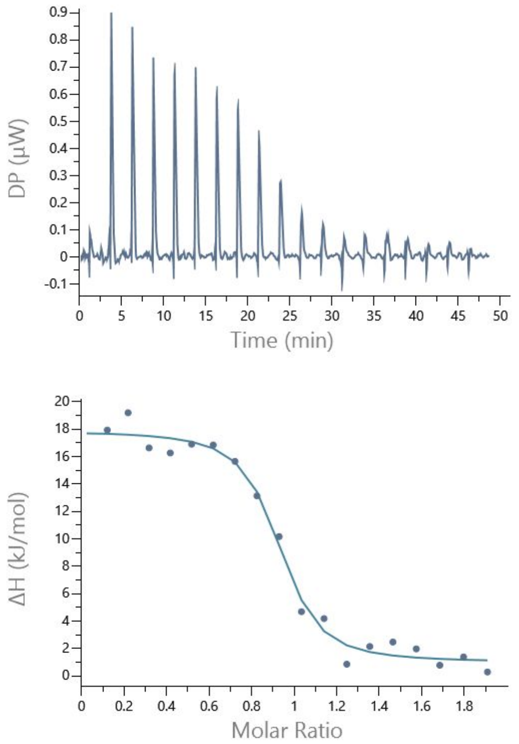

PabA wt + PabB E119D

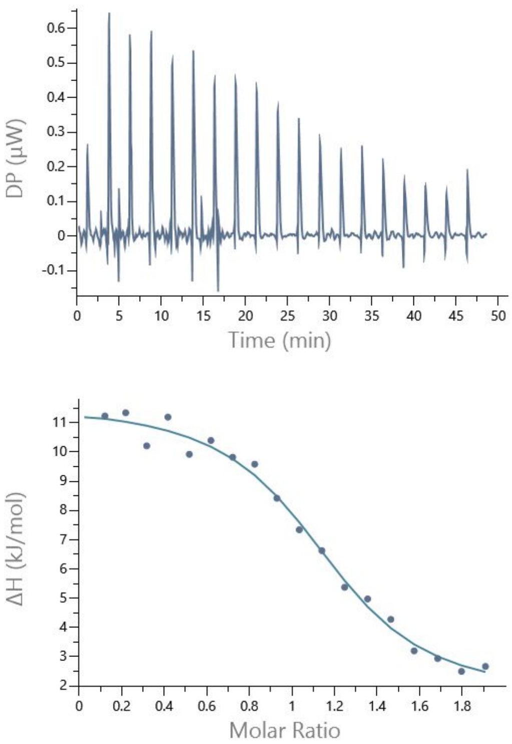

PabA wt + PabB E119R

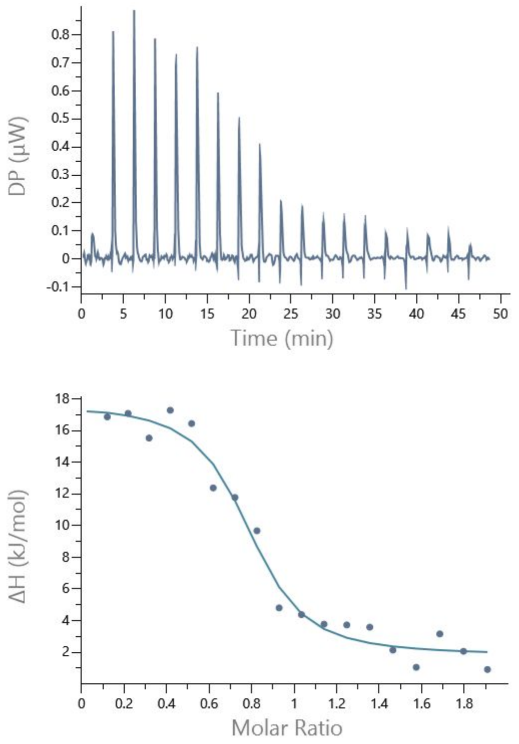

PabA P53A + PabB wt

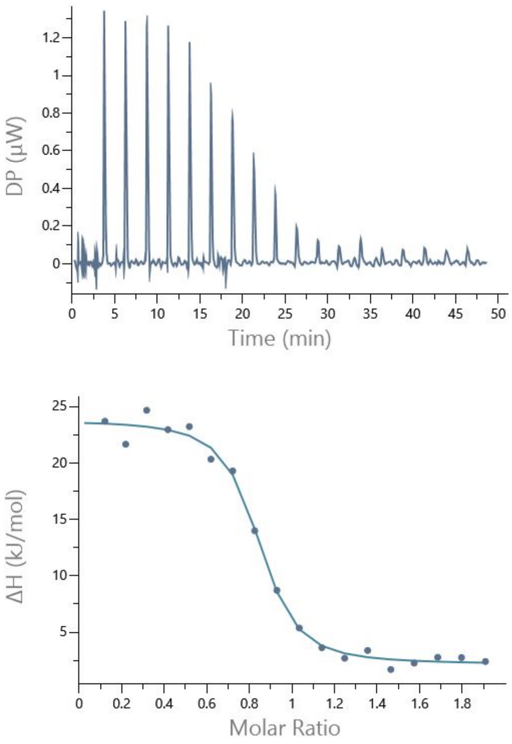

PabA C54G + PabB wt

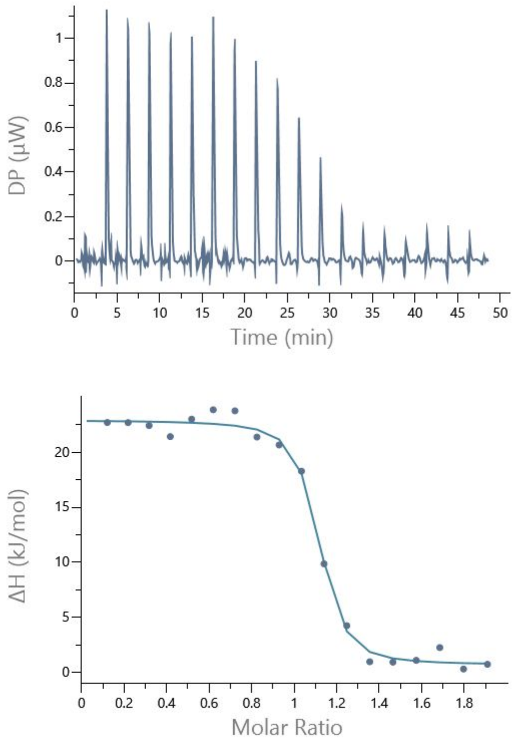

PabA C54A + PabB wt

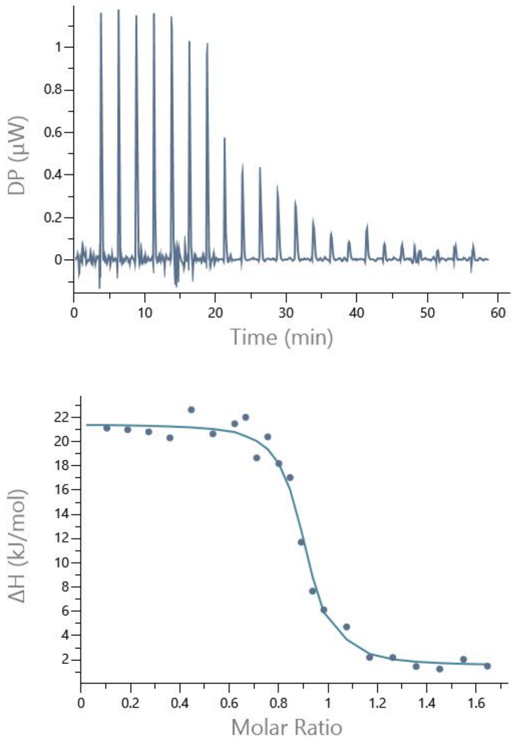

PabA H128A + PabB wt

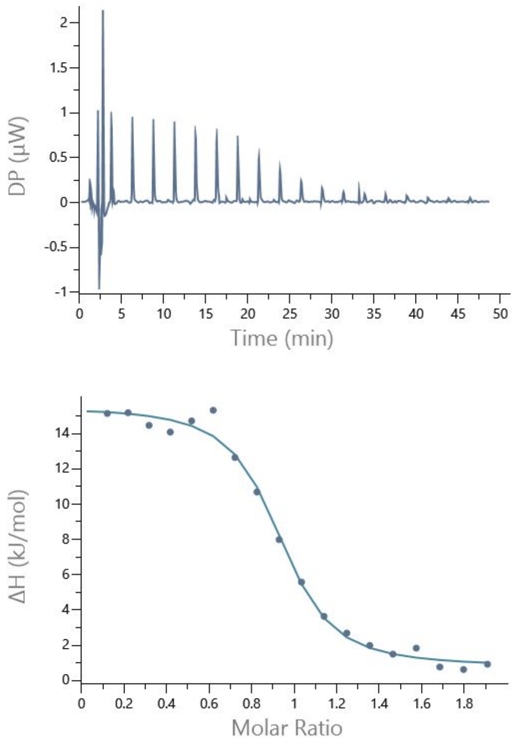

PabA wt + PabB G207P

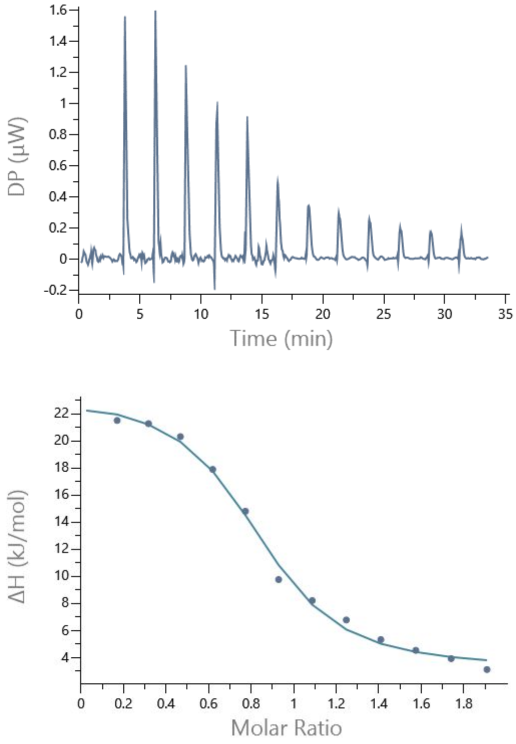

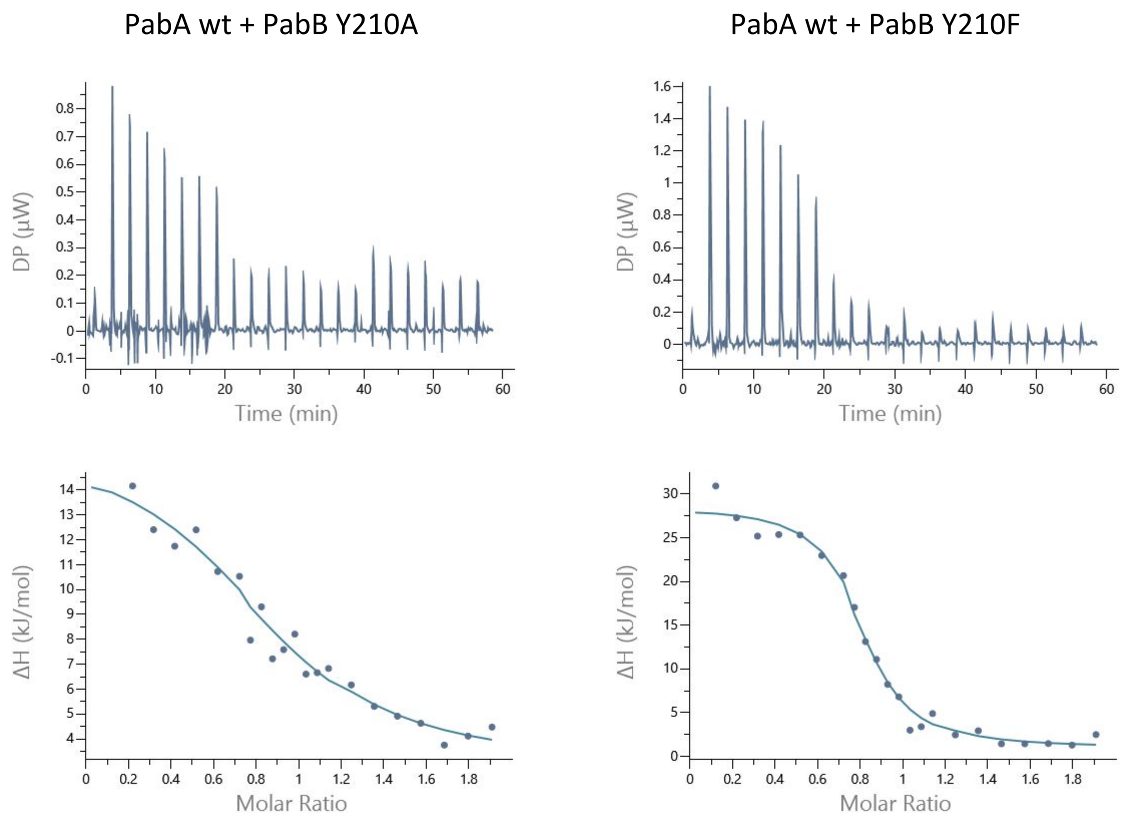

Supplementary Figure 14. ITC curves to analyze PabA/PabB complex formation. Experiments were performed using a MicroCal PEAQ-ITC instrument (Malvern) at 25 °C in 50 mM Tris/HCl pH 7.5, 50 mM KCl and 5 mM $MgCl_2$. A total of 19 or 23 injections of 1 or 2 µl 250 µM PabB were titrated to 300 µl of 25 µM PabA. Enthalpy differences were obtained by integrating the heat pulses of each injection (upper graphs) and plotted against the PabB/PabA ratio (lower graphs). From the resulting isotherm, the binding stoichiometry N, dissociation constant K_D and thermodynamic parameters ΔH and ΔS were calculated. ADCS variant as indicated above each set of graphs. One ITC curve is shown exemplarily for each variant.

PabA wt + PabB wt

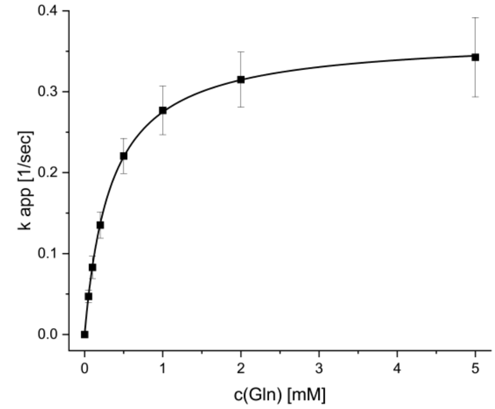

PabA wt + PabB wt -Cho

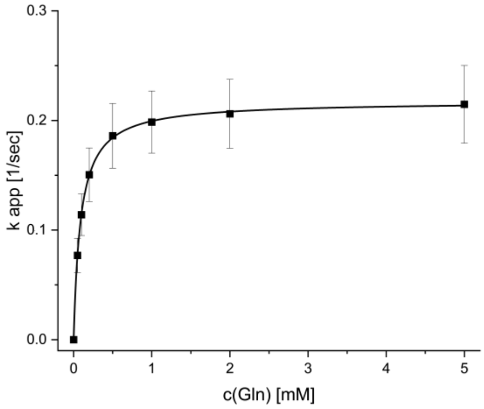

PabA wt

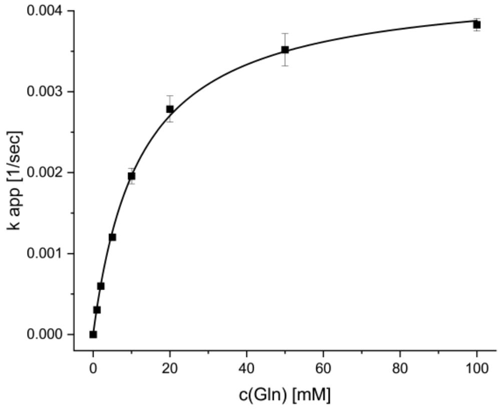

PabA Y127A + PabB wt

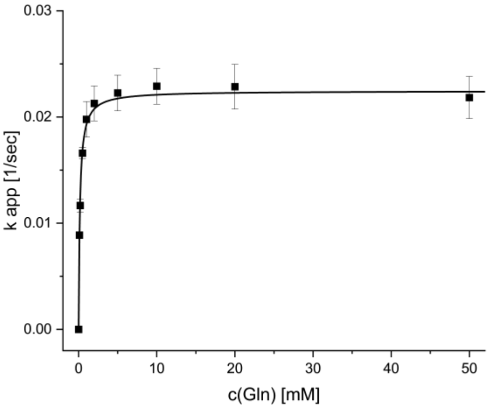

PabA S171A + PabB wt

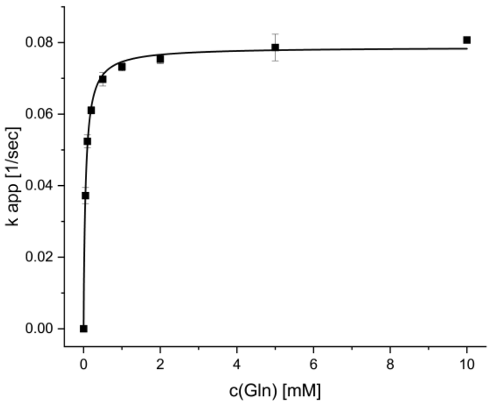

PabA [Y127A, S171A] + PabB wt

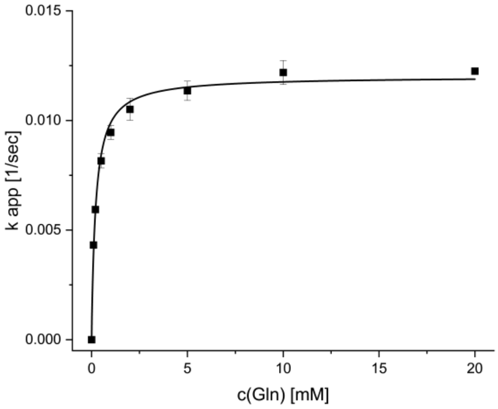

PabA wt + PabB D308A

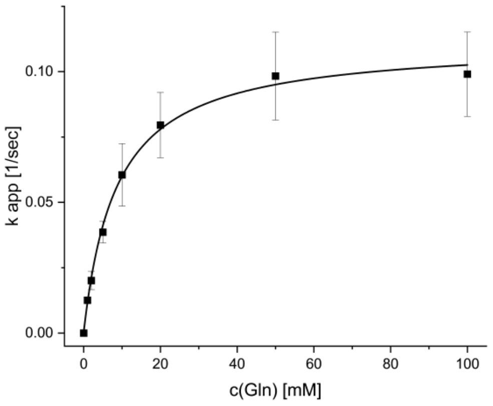

PabA wt + PabB D308N

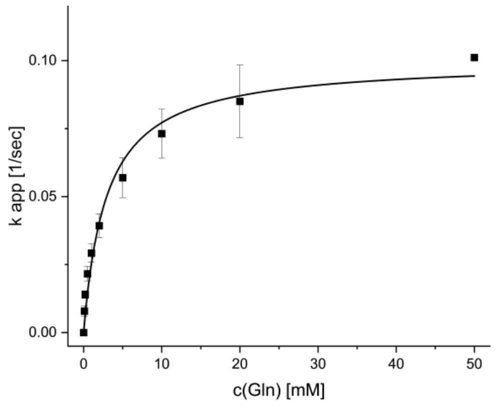

PabA wt + PabB D308E

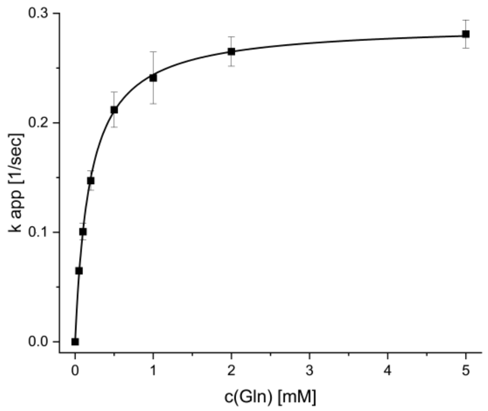

PabA wt + PabB R311A

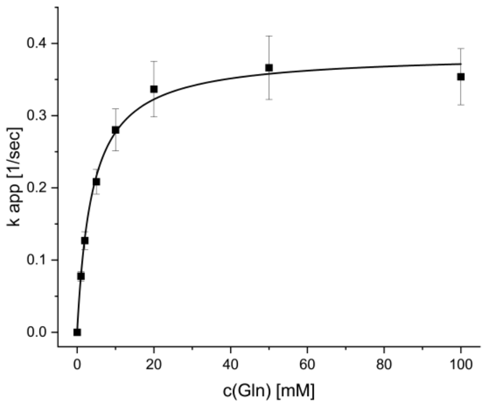

PabA R30E + PabB wt

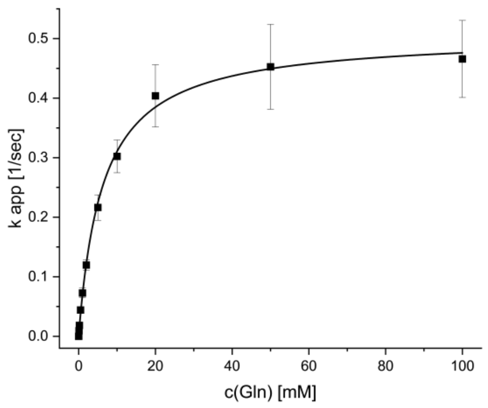

PabA wt + PabB E119D

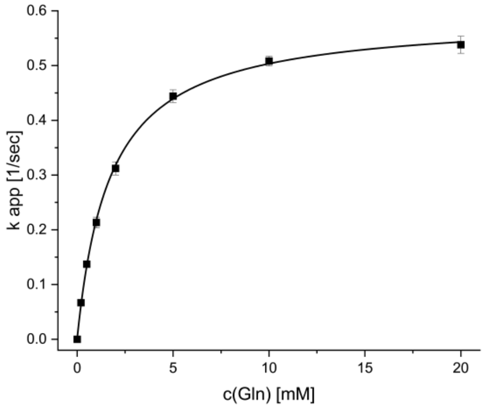

PabA wt + PabB E119R

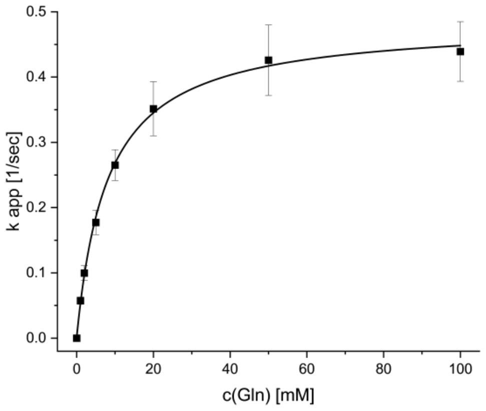

PabA P53A + PabB wt

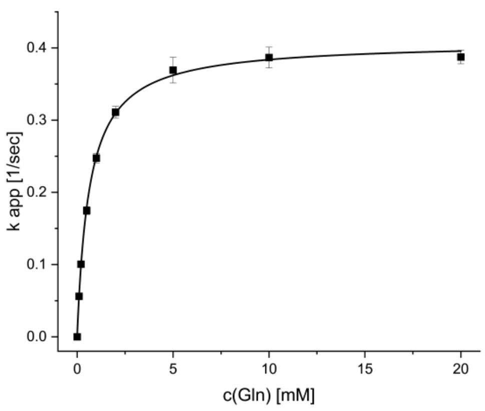

PabA C54G + PabB wt

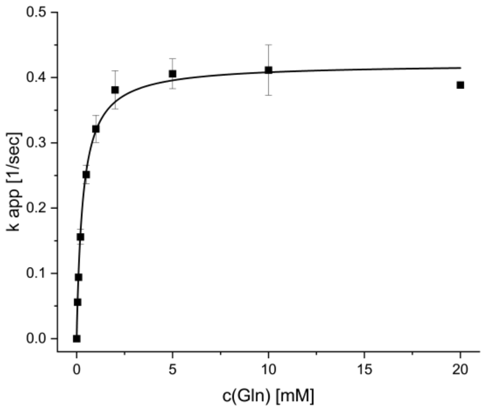

PabA C54A + PabB wt

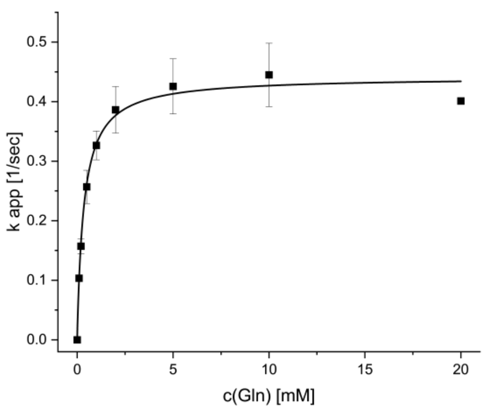

PabA H128A + PabB wt

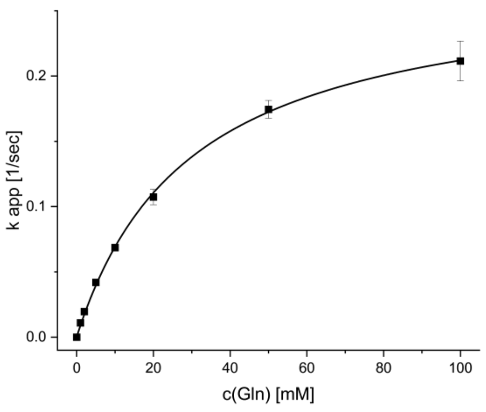

PabA wt + PabB G207P

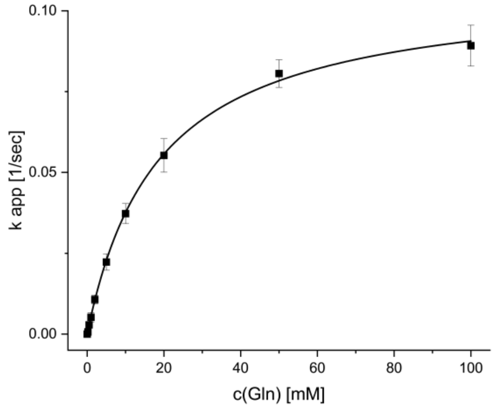

PabA wt + PabB Y210A PabA wt + PabB Y210F

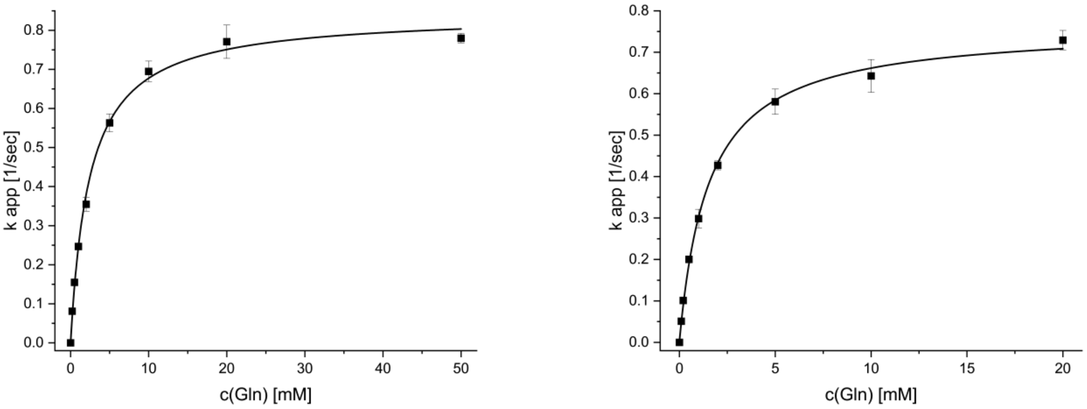

Supplementary Figure 15. Steady-state enzyme kinetic saturation curves to analyze PabA glutaminase activity. Experiments were performed in 50 mM Tricine pH 8, 5 mM $MgCl_2$, 150 mM KCl, 1 mM DTT, 10 mM NAD^+, 1 mg/ml GDH, 200 µM Cho and varying Gln concentrations at 25 °C in 96-well plates using a plate reader (Tecan Infinite M200 Pro). k_{app} values are plotted against the concentration of Gln, the applied hyperbolic fit is shown as a black curve. The data points and standard deviations are based on a biological duplicate or triplicate, each with two technical replicates. ADCS variant as indicated above each graph.

Reference List

1. Roux, B. & Walsh, C.T. p-aminobenzoate synthesis in Escherichia coli: kinetic and mechanistic characterization of the amidotransferase PabA. *Biochemistry* **31**, 6904-10 (1992).
2. Lee, Y.M. & Lim, C. Physical basis of structural and catalytic Zn-binding sites in proteins. *J Mol Biol* **379**, 545-53 (2008).
3. Li, Q.A., Mavrodi, D.V., Thomashow, L.S., Roessle, M. & Blankenfeldt, W. Ligand binding induces an ammonia channel in 2-amino-2-desoxyisochorismate (ADIC) synthase PhzE. *J Biol Chem* **286**, 18213-21 (2011).
